# Sero-Prevalence and Genetic Diversity of Pandemic *V. parahaemolyticus* Strains Occurring at a Global Scale

**DOI:** 10.3389/fmicb.2016.00567

**Published:** 2016-04-22

**Authors:** Chongxu Han, Hui Tang, Chuanli Ren, Xiaoping Zhu, Dongsheng Han

**Affiliations:** ^1^Clinical Medical Examination Center, Northern Jiangsu People's HospitalYangzhou, China; ^2^Experimental Research Center, Northern Jiangsu People's HospitalYangzhou, China

**Keywords:** *Vibrio parahaemolyticus*, multilocus sequence typing, pandemic clone, gastroenteritis, phylogenetic analysis

## Abstract

Pandemic *Vibrio parahaemolyticus* is an emerging public health concern as it has caused numerous gastroenteritis outbreaks worldwide. Currently, the absence of a global overview of the phenotypic and molecular characteristics of pandemic strains restricts our overall understanding of these strains, especially for environmental strains. To generate a global picture of the sero-prevalence and genetic diversity of pandemic *V. parahaemolyticus*, pandemic isolates from worldwide collections were selected and analyzed in this study. After a thorough analysis, we found that the pandemic isolates represented 49 serotypes, which are widely distributed in 22 countries across four continents (Asia, Europe, America and Africa). All of these serotypes were detected in clinical isolates but only nine in environmental isolates. O3:K6 was the most widely disseminated serotype, followed by O3:KUT, while the others were largely restricted to certain countries. The countries with the most abundant pandemic serotypes were China (26 serotypes), India (24 serotypes), Thailand (15 serotypes) and Vietnam (10 serotypes). Based on MLST analysis, 14 sequence types (STs) were identified among the pandemic strains, nine of which fell within clonal complex (CC) 3. ST3 and ST305 were the only two STs that have been reported in environmental pandemic strains. Pandemic ST3 has caused a wide range of infections in as many as 16 countries. Substantial serotypic diversity was mainly observed among isolates within pandemic ST3, including as many as 12 combinations of O/K serotypes. At the allele level, the *dtdS* and *pntA*, two loci that perfectly conserved in CC3, displayed a degree of polymorphism in some pandemic strains. In conclusion, we provide a comprehensive understanding of sero-prevalence and genetic differentiation of clinical and environmental pandemic isolates collected from around the world. Although, further studies are needed to delineate the specific mechanisms by which the pandemic strains evolve and spread, the findings in this study are helpful when seeking countermeasures to reduce the spread of *V. parahaemolyticus* in endemic areas.

## Introduction

*Vibrio parahaemolyticus*, an organism with a high genetic diversity, has emerged as a pathogen causing acute gastroenteritis with a worldwide distribution. Before 1996, *V. parahaemolyticus* infections usually exhibited a localized distribution and were linked to diverse serotypes (e.g., O2:K3, O3:K6, O4:K8) (Wong et al., 2000). In February 1996, the pandemic O3:K6 serotype emerged, resulting in an inexplicable increase of *V. parahaemolyticus* gastroenteritis in Kolkata city, India (Okuda et al., 1997). This unique serotype subsequently quickly spread into coastal regions of southern Asia (Bag et al., 1999), America (Martinez-Urtaza et al., 2004), Africa (Ansaruzzaman et al., 2005), and Europe (Martinez-Urtaza et al., 2005) and caused numerous outbreaks within a few years (Okuda et al., 1997; Chowdhury et al., 2000a; Nair et al., 2007). Such widespread occurrence of a single serotype of *V. parahaemolyticus* had not been previously reported.

All the pandemic O3:K6 strains share the following specific genetic markers: positivity for the thermostable direct hemolysin(*tdh*) gene, negativity for the TDH-related hemolysin(*trh*) gene and positivity for a toxRS/*new* gene, which can be amplified via a specific PCR method known as “GS-PCR” (Matsumoto et al., 2000; Chao et al., 2011; de Jesús Hernández-Díaz et al., 2015). To our surprise, in recent years, some new serotypes [e.g., O4:K68, O1:K25, O1:KUT(untypable)] have been detected that exhibit identical genotypes and molecular profiles to the pandemic O3:K6 serotype (Chang et al., 2000; Bhuiyan et al., 2002). These serotypes may diverge from the pandemic O3:K6 serotype in alteration of the O and/or K antigens and are referred to as “serovariants” of the pandemic O3:K6 serotype (Chowdhury et al., 2000b; Matsumoto et al., 2000). Currently, all of the pandemic serotypes are grouped as belonging to the “O3:K6 pandemic clone.” Through 2007, a total of 22 serotypes had been reported to belong to this clone (Nair et al., 2007).

Many surveys have shown that pandemic *V. parahaemolyticus* serovariants can be identified not only in clinical samples (Li et al., 2014; Pazhani et al., 2014; Ueno et al., 2016), but also in seafood and other environmental samples (Arakawa et al., 1999; Vuddhakul et al., 2000; Deepanjali et al., 2005; Quilici et al., 2005; Chao et al., 2009; Caburlotto et al., 2010), indicating that the pandemic strains have established ecological niches in many regions, resulting in a heightened perception of the threat to the public health of the local population. An accurate description of the distribution and spread of the pandemic strains is important for understanding the epidemiology of this pathogen and preventing outbreaks and sporadic illnesses. However, after G. Balakrish Nair and colleagues reviewed the global dissemination of pandemic *V.parahaemolyticus* serotype O3:K6 and its serovariants in 2007 (Nair et al., 2007), few studies have specifically for the pandemic *V. parahaemolyticus* on a global scale, especially concerning the worldwide dissemination of environmental strains. Therefore, it would be beneficial to integrate and update the available scientific data on pandemic *V. parahaemolyticus*.

The establishment of a multilocus sequence typing (MLST) scheme for *V. parahaemolyticus* has enhanced our knowledge of the population structure and genetic diversity of *V. parahaemolyticus* (Gonzalez-Escalona et al., 2008). Previous studies based on MLST assay have shown that the increasing prevalence of clonal complex 3 (CC3) has become an ongoing public health concern (Gonzalez-Escalona et al., 2008; Haendiges et al., 2014; Han et al., 2015), and most pandemic strains have been identified as belonging to CC3 (Chen et al., 2016). Thus, clarifying the genetic diversity among the pandemic strains will aid in the selection of preventative strategies targeting pandemic strain infections.

In this study, we collected data on pandemic strains mainly from the pubMLST database (http://pubmlst.org/vparahaemolyticus) and previous studies, in an effort to generate a comprehensive overview of the spread of clinical and environmental pandemic *V. parahaemolyticus* strains occurring over wide geographic areas since the emergence of this clone. Furthermore, through MLST phylogenetic analysis, we determined the genetic diversity of the pandemic clone to provide a holistic understanding of the microevolution of pandemic strains.

## Materials and methods

### Datasets utilized in the present study

A total of 267 representative clinical and environmental *V. parahaemolyticus* isolates with pandemic genetic marks (toxRS/*new*+, *tdh*+, and *trh*−) were selected as the research subject of this study, among which 263 isolates came from the literature and four from the pubMLST database (http://pubmlst.org/vparahaemolyticus/). To identify relevant publications, we conducted a comprehensive search of the US National Library of Medicine PubMed database and the Elsevier, Springer, and China National Knowledge Infrastructure databases for all relevant studies using combinations of the following terms: “*Vibrio parahaemolyticus*,” “pandemic clone,” “pandemic strains,” and “O3:K6 clone” (until July 1, 2015). Additional eligible studies were identified from references cited in the relevant articles. The full text of each potentially relevant paper was scrutinized and a total of 263 isolates with pandemic genetic marks (toxRS/*new*+, *tdh*+, and *trh*−) were finally extracted from 39 papers. Details on the individual isolates are summarized in Additional file 1: Table S1.

### Multilocus sequence typing analysis

To determine the genetic diversity of the pandemic strains through MLST analysis, another file containing 185 isolates were analyzed (see Additional file 2: Table S2). These isolates included 95 pandemic isolates from Table S1 and 90 strains that are non-pandemic but belong to CC3 from the pubMLST database. The sequence types (STs) of the 185 isolates were compared using eBURST V3 (http://eburst.mlst.net/) (Feil et al., 2004). Additionally, a “population snapshot” analysis of the entire *V. parahaemolyticus* population was also implemented based on the total pubMLST dataset, which illustrated the population differentiation of the whole population. Clonal complexes were defined conservatively as a cluster of STs in an eBURST diagram, in which all STs were linked as single-locus variants (SLVs, two STs differing from each other at a single locus) to at least one other ST (Feil et al., 2004). The singleton STs corresponded to STs differing from all the others by three or more of the seven loci (Esteves et al., 2015).

### Genetic diversity and phylogenetic analysis

The diversity of the seven loci in the pandemic isolates was revealed by DnaSP V5 (http://www.ub.edu/dnasp/) with respect to the following parameters: the number of alleles, number (%) of polymorphic sites, nucleotide diversity (per site) and Tajima's D value. The purpose of Tajima's D test is to distinguish housekeeping genes evolving randomly (“neutrally”) vs. those evolving under a non-random process (Tajima, 1989). A *P* > 0.05 indicates that the target gene is evolving randomly and that mutations in the gene have no effect on the fitness and survival of an organism (Tajima, 1989; Ferreira et al., 2008). A minimum-evolution (ME) tree for the concatenated sequences of each ST of the 185 isolates was generated using Mega 5 software with the Kimura two-parameter model to estimate genetic distances. The statistical support for the nodes in the ME tree was assessed through 1000 bootstrap resamplings.

## Results

### Global spread of pandemic serovariants

According to a detailed review, a total of 49 pandemic serotypes from 22 countries across four continents (Asia, Europe, America, and Africa) were identified. All of these serotypes were detected in clinical isolates but only nine in environmental isolates. O3:K6 was the most widely disseminated serotype, and patients in all 22 countries had been infected with this subtype at some point in time. O3:KUT was the second most widely distributed serotype. Several serotypes, such as O1:K25, O1:KUT, and O4:K68, also exhibited multi-country distributions but were mainly restricted to Southeast Asia (Table 1).The sources of environmental pandemic isolates were diverse, mainly including shellfish, oyster, clam, and shrimp, sediment and seawater samples collected in nine countries (see in Additional file 1: Table S1).

**Table 1:** **Presence of clinical and environmental pandemic serovariants of ***V. parahaemolyticus*** occurring at a global scale**.

**Serotype**	**Country (year of isolation)**
**CLINICAL(49 SEROTYPES):**	
O1:K25	Bangladesh(1999), China(1998, 2005–2012), India(2004–2010), Thailand(1999–2010), Vietnam(1998–1999), Japan (1998)
O1:K26	China(2007)
O1:K30	India(2011)
O1:K33	India(2002)
O1:K36	China(2006–2012)
O1:K38	India(2001)
O1:K41	Thailand(1998–1999, 2002), Vietnam(1998–1999)
O1:K5	China(2007, 2009)
O1:K56	China(2008), India(2011), Vietnam(1998–1999)
O1:KUT^*^	Bangladesh(1998, 2000), China(2003,2005–2012), India(1998, 2001), Peru(2005, 2007), Thailand(2001–2010)
O10:K60	China(2010–2012), Japan(2013), India(2012)
O10:KUT	Mexico(2004–2010)
O11:K36	China(2007)
O2:K3	China(2010–2012), Thailand(2000), India(2002)
O2:K4	India(2005)
O3:K25	China(2007)
O3:K29	China(2007), Thailand(2002–2003), Mexico(2011–2013)
O3:K3	China(2010–2012)
O3:K30	Peru(2007)
O3:K46	Thailand(2001, 2004)
O3:K5	India(2004)
O3:K58	Peru(1998–1999)
O3:K59	Chile(2007)
O3:K6	Angola(1999), Bangladesh(1998), Brazil(2002), Chile(1998, 2007), China(1996–2012), Ecuador(1999), France(2004), India(1996), Indonesia(1997), Italy(2007–2008), Japan(1996, 1998), Korea(1997–1998), Laos(1997), Mexico(2004–2013), Mozambique(2004), Peru(1996–2003), Russia(2012), Singapore(1996,1998), Thailand(1996–1997,2000–2010), Vietnam(1997), Spain(2004), USA(1998,2012)
O3:K6,59	Chile(2007)
O3:K68	China(2006), Peru(1998)
O3:K75	Vietnam(1998–1999)
O3:K8	China(2009–2011)
O3:KUT	Brazil(2002), China(2010–2012), India(2003–2004), Peru(2007), Spain(2004), Mexico(2004–2010), Thailand(2004,2006–2010)
O4:K10	India(2004)
O4:K12	Chile(2004), Thailand(1998–1999), Mexico(2004–2010), Vietnam(1998–1999)
O4:K13	India(2010)
O4:K25	India(2005)
O4:K4	India(2004), Thailand(2005)
O4:K48	China(2005–2008)
O4:K55	India(2001)
O4:K68	Bangladesh(1998), China(1999, 2003, 2005–2012), India(1998–1999), Mozambique(2004), Singapore(1998), Vietnam(1998), Thailand(1999–2001, 2003–2005)
O4:K8	China(2006–2010), Thailand(2006–2010), Vietnam(1998–1999)
O4:K9	Thailand(2006–2010)
O4:KUT	China(2006–2007), India(2009), Vietnam(1998–1999)
O5:K17	India(2002)
O5:K25	India(2002)
O5:K68	China(2007–2012), Norway(2002)^#^
O5:KUT	China(2010–2012), India(2004), Vietnam(1998–1999), Thailand(2003)
O6:K18	China(2005), Singapore(1998)
O8:K21	India(2006)
OUT:K6	Peru(1998)
O10:KUT	Mexico(2011–2013)
OUT:KUT	China(2007), India(2003–2004), Mexico(2004–2013)
**ENVIRONMENT (9 SEROTYPES):**	
O3:K6	Chile(2008–2009), China(2005–2008), Italy(2007), France(1997, 1998, 1999), Mexico(2004–2013), the UK(2012), Bangladesh(2007), Japan(1998, 2000, 2001), India(2002)
O3:KUT	Mexico(2011–2013)
O4:K48	China(2005–2008)
O1:KUT	China(2005–2008)
O1:K25	Japan(2001)
O4:K10	Mexico(2012)
O4:K68	Japan(2001)
O10:KUT	Mexico(2004–2010)
OUT:KUT	Mexico(2004–2010)

* UT, untyped.

# There was no sufficient evidences to determine the isolate of pandemic O5:K68 was really collected from Norway in 2002.

**Figure 1:**
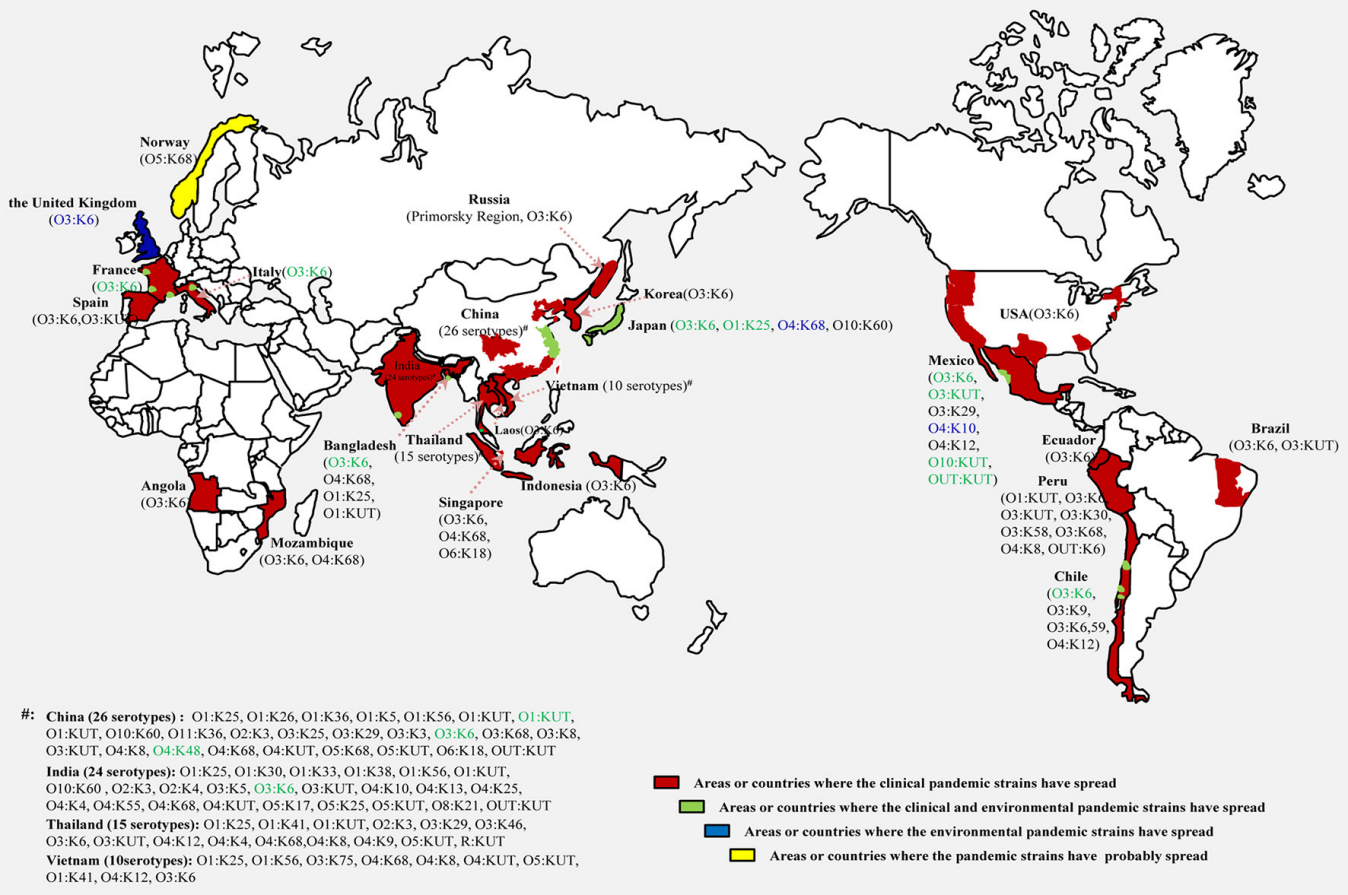
**Map showing the dissemination of clinical and environmental pandemic serovariants of ***V. parahaemolyticus*** occurring at a global scale**. Serotypes identified in clinical isolates (black), environmental isolates (blue) and both in clinical and environmental isolates (green) are marked respectively.

A comprehensive map of the dissemination of the clinical and environmental pandemic serotypes on a global scale was generated (Figure 1). The serotypes of the pandemic clone were highly abundant and variable in coastal regions of China, India, Thailand and Vietnam. It was notable that most of the environmental pandemic serotypes present in a certain country were also detected in patients from that country. O3:K6 was the typical serotype. Four environmental serotypes (O3:K6, O3:KUT, O10:KUT, and OUT:KUT) in Mexico were also found spread in its local population.

### Widely dispersed clones of *V. parahaemolyticus* and genetic differentiation of the pandemic isolates

**Figure 2:**
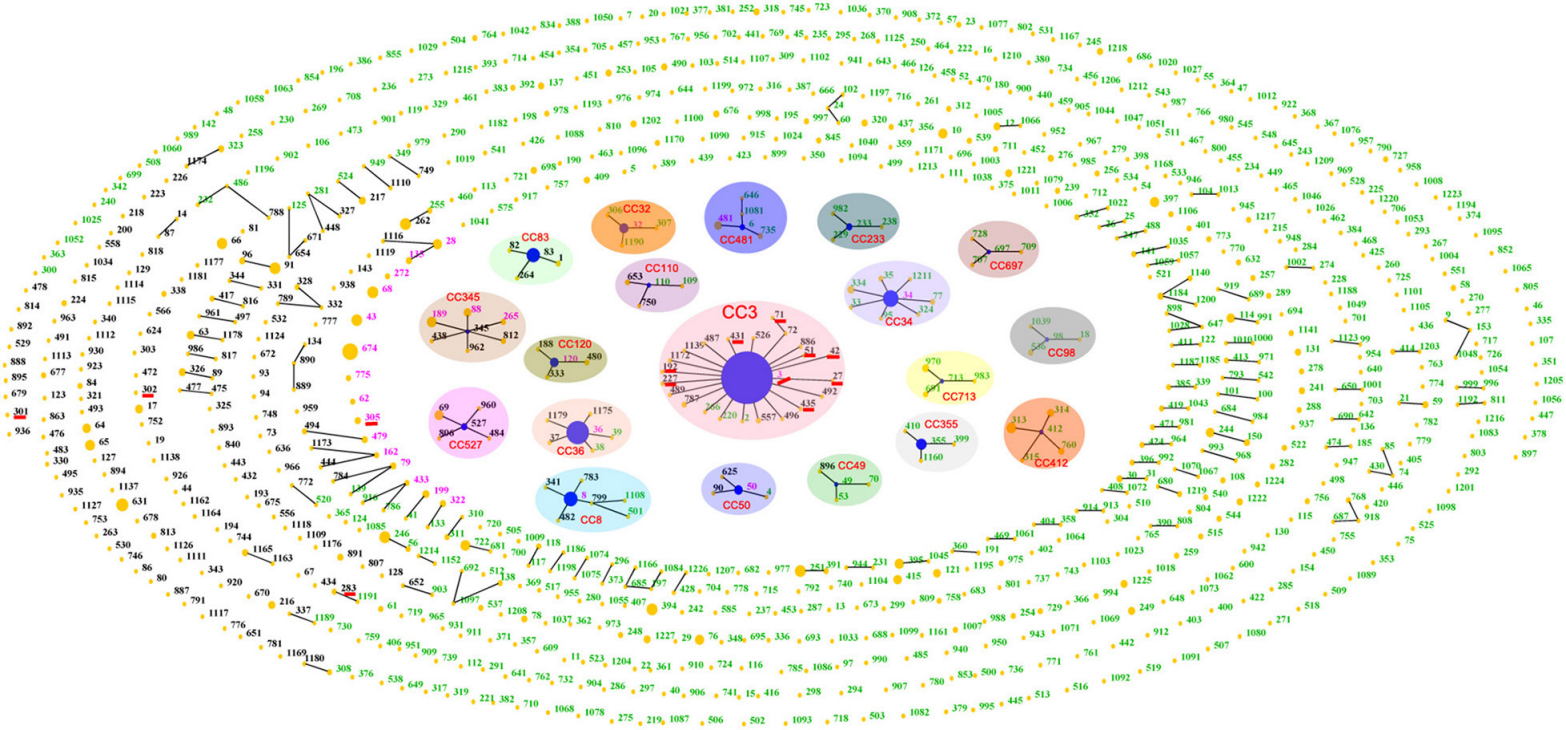
**“Population snapshot” showing the clonal diversity of ***V. parahaemolyticus*** based on data archived in public database**. Green numbers indicate STs typed in environmental isolates; black numbers represent clinical STs; and pink numbers are STs that found in both clinical and environmental collections. Nineteen clonal complexes are indicated with separate shades. STs that are SLVs of each other are connected with black lines. STs associated with pandemic spreading are underlined in red.

Until August 2015, a total of 954 STs had been identified in the *V. parahaemolyticus* pubMLST database, approximately two-thirds of which were detected in environmental isolates, while less than one-third came from clinical isolates, and only 26 were present both in environmental and clinical isolates. The total population displayed 19 CCs as well as some doublets and numerous singletons (Figure 2). CC3 was the most prevalent CC, being comprised of 18 STs with no less than 15 serotypes (Table 2).

**Table 2:** **Sequence types, allele profiles, and geographic locations of CC3 and pandemic ***V. parahaemolyticus*** clone**.

**Clone**		**ST**	**Allele types**							**Collection countries (serotypes)**	
**CC3**	**Pandemic**		***dnaE***	***dtdS***	***gyrB***	***pntA***	***pyrC***	***recA***	***tnaA***	**clinical**	**environment**
**Yes**	No	2	**11**^&^	4	4	29	4	19	22	−	China(O3:K6)
**Yes**	No	72	3	4	4	29	4	**4**	22	Thailand(O3:KUT)	−
**Yes**	No	220	3	4	4	29	4	**15**	22	−	China(O3:K6)
**Yes**	No	266	3	4	4	29	4	19	**78**	−	China(O1:K33)
**Yes**	Unknown	557	3	4	4	29	4	19	**152**	China(Unknown)	−
**Yes**	Unknown	787	3	4	4	29	**48**	19	22	China(O4:K68)	−
**Yes**	Unknown	886	**51**	4	4	29	4	19	22	China(Unknown)	−
**Yes**	Unknown	1139	3	4	**415**	29	4	19	22	Mexico(O3:K6)	−
**Yes**	Unknown	1172	3	4	**23**	29	4	19	22	Chile(Unknown)	−
**Yes**	**Yes**	3	3	4	4	29	4	19	22	^*^	^#^
**Yes**	**Yes**	27	**17**	4	4	29	4	19	22	Korea(O3:K6)	−
**Yes**	**Yes**	42	**22**	4	4	29	4	19	22	USA(O3:K6)	−
**Yes**	**Yes**	51	**29**	4	4	29	4	19	22	Bangladesh(O3:KUT)	−
**Yes**	**Yes**	71	3	4	4	29	4	**4**	**20**	Ecuador(O3:K6)	−
**Yes**	**Yes**	192	3	4	**126**	29	4	19	22	China(O1:K26)	−
**Yes**	**Yes**	227	3	4	4	29	**22**	19	22	China(Unknown)	−
**Yes**	**Yes**	431	3	4	**225**	29	4	19	22	China(Unknown)	−
**Yes**	**Yes**	435	3	4	4	29	4	**31**	22	China(O3:K6)	−
No	**Yes**	283	**27**	**84**	**127**	**139**	**54**	**124**	**37**	China(O4:K8)	−
No	**Yes**	301	**140**	**167**	**136**	**151**	**50**	**135**	**17**	China(O4:KUT)	−
No	**Yes**	302	**27**	**106**	**127**	**152**	**54**	**124**	**101**	China(O4:KUT)	−
No	**Yes**	305	3	**147**	4	**93**	4	19	22	China(O1:K25)	China(O1:KUT)
No	**Yes**	672	**1**	4	**147**	29	4	19	22	China(O3:K6)	−

* *Bangladesh(O1:K25,O1:KUT,O3:K6,O4:K68), Chile(O3:K6), China(O1:K25, O1:K36,O1:KUT,O11:K36,O3:K25,O3:K6,O3:K68,O4:K68), Ecuador(O3:K6), India(O1:KUT,O3:K6,O4:K68), Indonesia(O3:K6), Japan(O1:K25,O3:K6), Korea(O3:K6), Mexico(O3:K6), Mozambique(O3:K6,O4:K68), Norway(O5:K68), Peru(O1:KUT,O3:K30,O3:K58,O3:K6,O3:KUT), Singapore(O3:K6,O4:K68), Spain(O3:K6), Thailand(O1:K25,O3:KUT,O4:K68), USA(O3:K6)*.

# *Chile(O3:K6), China(O1:KUT). These isolates belong to the pandemic clone. Others can't be determined as pandemic isolates but typed as ST3 were not listed here, see them in Additional file 2: Table S2*.

& *Compared with ST3, the changed allele types in other STs in bold*.

**Figure 3:**
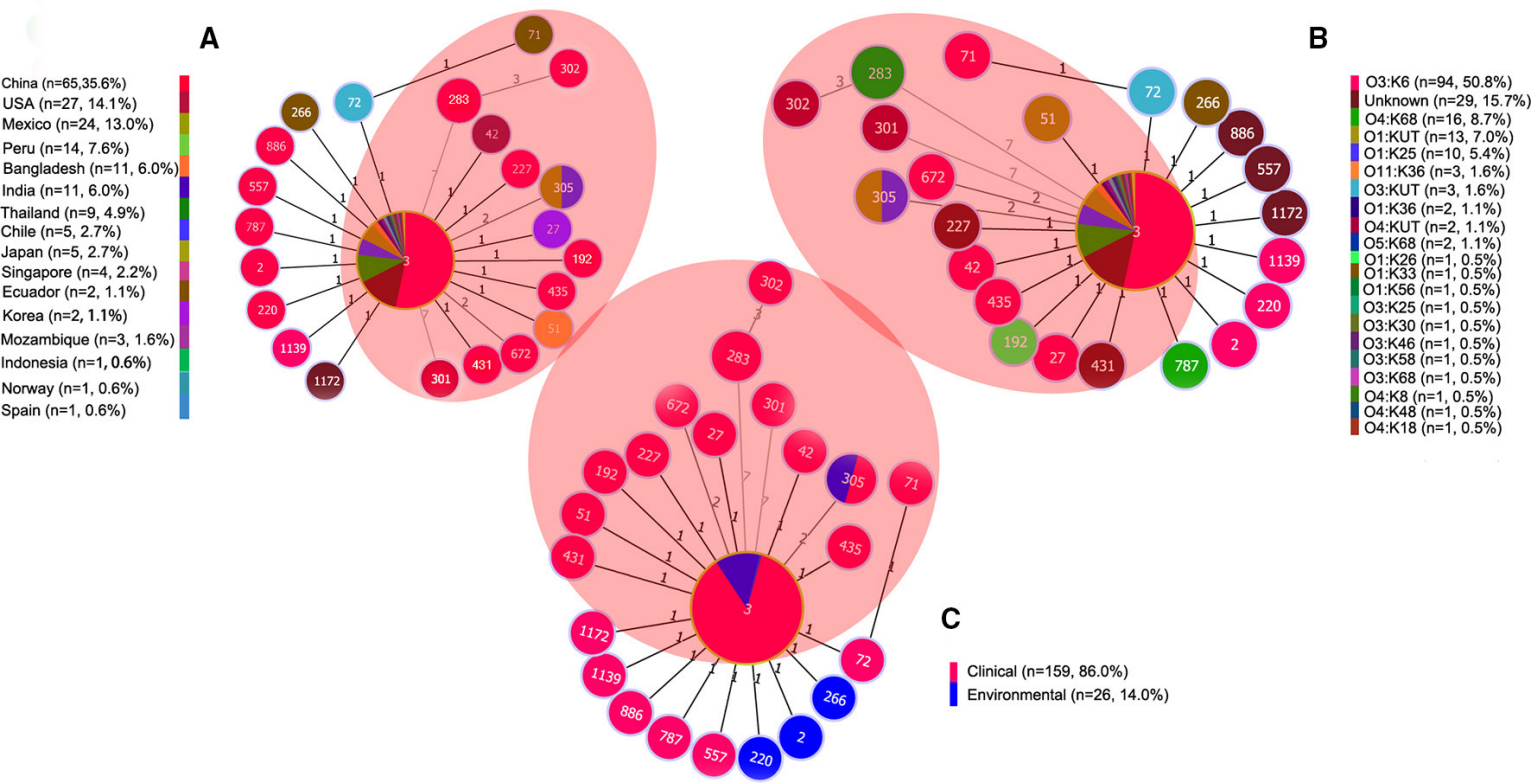
**goeBURST full MST of the STs consisting of 185 representative isolates associated with CC3 and/or the pandemic clone. (A)** Geographical distribution, **(B)** Serotype distribution, **(C)** Source distribution. The sizes of the circles vary according to the frequency of the ST. The number of different alleles is presented between STs connected via a line. ST3 and its SLVs (one allele diversity) form the CC3. STs in the shaded area are associated with the pandemic clone. “n” in the figure legend indicates the number of isolates.

After thoroughly analyzing the sequence data for the 185 isolates (in Additional file 2: Table S2), we found that the pandemic strains exhibited 14 STs, only two of which (ST3 and ST305) had ever been identified in environmental samples (Figure 3C). China was the country with the most pandemic STs (10 STs). Nine of these 14 pandemic STs could be classified into CC3, among which, ST3 was the only pandemic ST that had caused a wide range of infections in as many as 16 countries (Table 2, Figure 3A). ST305 and ST672 were DLVs of ST3 but were not members of CC3 because there was no ST in CC3 could act as their SLV. The other three STs (ST283, ST301, and ST302) originating from coastal areas of China were identified as singletons with no relationship to CC3.

### The association between pandemic STs and serotypes

The Minimum spanning tree of the 14 pandemic STs resulting from the MLST analysis showed a substantial serotypic diversity among isolates within ST3, but not among isolates of the other 13 STs (Figure 3B). Specifically, pandemic ST3 comprised isolates of 12 serotypes (O1:K25, O1:K36, O1:KUT, O3:K6, O3:K25, O3:K30, O3:K58, O3:K68, O3:KUT, O4:K68, O5:K68, and O11:K36). ST305 included isolates belonging to O1:K25 and O1:KUT serotypes. The remaining STs were consisted of a single serotype, respectively (Table 2, Figure 3B). From another perspective, the pandemic O3:K6 serotype was shared by six different STs (ST3, ST27, ST42, ST71, ST435, and ST672). Other serotypes were clustered in no more than two different pandemic STs, respectively (Table 2).

### Genetic diversity of the pandemic isolates

The data on the nucleotide and allelic diversity of the pandemic isolates are summarized in Table 3. The highest percentage of polymorphic sites was detected in *dtdS* (5.46%). Nucleotide diversity ranged from 0.01082 (*pyrC*) to 0.02926 (*dtdS*). *dtdS* and *pntA* were perfectly conserved in CC3 (the allele types were *dtdS4* and *pntA29*), but in the pandemic isolates, five different alleles were detected for each of the two genes; the number of SNPs was 25(5.46%) for *dtdS* and 10(2.33%) for *pntA* (Table 3).

**Table 3:** **Sequence analysis of the seven loci studies for the isolates of CC3 and pandemic clone**.

**Locus**	**Fragment size**	**No of alleles**		**Number (%) of polymorphic sites**		**Nucleotide diversity (per site)**		**Tajima's D (***P***-Value)**	
		**CC3**	**Pandemic Clone**	**CC3**	**Pandemic Clone**	**CC3**	**Pandemic Clone**	**CC3**	**Pandemic Clone**
*dnaE*	557	6	7	18 (3.23)	19 (3.41)	0.01352	0.01368	−0.27603 (*P* > 0.10)	−0.09825 (*P* > 0.10)
*gyrB*	592	5	6	10(5.92)	18(3.04)	0.00676	0.01261	−1.19267 (*P* > 0.10)	−0.32862 (*P* > 0.10)
*recA*	729	4	5	28(3.84)	26(3.57)	0.01920	0.02058	−0.86044 (*P* > 0.10)	1.50445 (*P* > 0.10)
*dtdS*	458	1	5	−	25(5.46)	−	0.02926	−	0.86832 (*P* > 0.10)
*pntA*	430	1	5	−	10 (2.33)	−	0.01209	−	0.59633 (*P* > 0.10)
*pyrC*	493	3	4	8(1.62)	10 (2.03)	0.01082	0.01082	−	−0.22234 (*P* > 0.10)
*tnaA*	423	4	5	11 (2.60)	15 (3.55)	0.01300	0.01608	−0.83741 (*P* > 0.10)	−0.40617 (*P* > 0.10)

*Four or more suquences are needed to compute the Tajima's test*.

### Phylogenetic analysis of pandemic isolates

**Figure 4:**
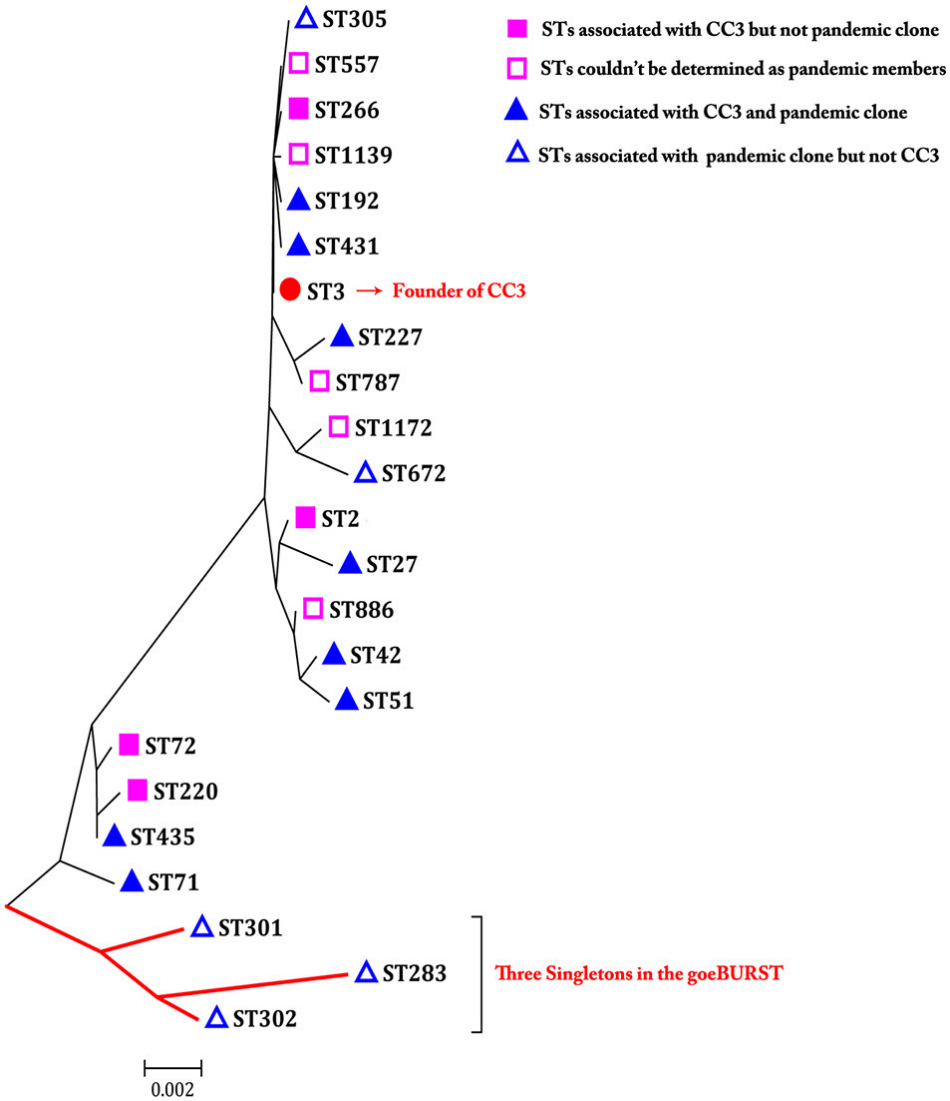
**A minimum evolutionary tree built using the concatenated sequences of the seven loci of each ST associated with pandemic clone and/or CC3**. The scale bar represents the evolutionary distance.

Phylogenetic analysis may provide a better resolution and elucidate some phylogenetic relationships among CCs or singletons that are not observed or resolved using goeBURST. Therefore, an ME tree representing the concatenated sequences of the seven housekeeping gene fragments in the 185 isolates is shown in Figure 4. In the goeBURST analysis, five pandemic STs (ST305, S672, ST301, ST302, and ST283) were not grouped into CC3. However, in the ME tree analysis, ST305 and ST672 were clustered together with STs of CC3, and only ST301, ST302, and ST283 exhibited relatively greater evolutionary distances from STs in CC3. In fact, the number of SNPs in the seven alleles of these last STs was greater than in ST305 and ST672 when compared with the STs of CC3.

## Discussion

In previous studies, we successfully made extensive descriptions of strains from a global clinical collection and from Chinese patients, respectively, exhibiting a highly degree of genetic diversity and a complicated population structure of *V. parahaemolyticus* in general (Han et al., 2014, 2015). In this study, we elucidated the sero-prevalence and genetic differentiation of the pandemic clone, which has becoming an emerging public health concern (Martinez-Urtaza et al., 2010; Velazquez-Roman et al., 2012, 2014; Powell et al., 2013; Li et al., 2014; Pazhani et al., 2014). The results will be useful in uncovering the microevolution relationships among pandemic *V. parahaemolyticus* strains. Serotyping is the primary basis of the classification of *V. parahaemolyticus* strains. Pandemic strains exhibit rapidly changing their serotypes (Nair et al., 2007). From1996 to 2007, 22 pandemic serotypes were identified (Nair et al., 2007). In the present study, as many as 49 serotypes identified to date in investigations conducted by different laboratory groups around the world could be confirmed as being associated with the pandemic clone.

Several lines of evidence have been presented in support of the hypothesis that these new serotypes might have emerged from the pandemic O3:K6 strains through replacement of the putative O and K antigen gene clusters (Okura et al., 2008; Harth et al., 2009). In the present study, as many as 12 combinations of O/K serotypes were grouped in pandemic ST3, demonstrating a remarkably high degree of serotypic diversity among the pandemic isolates and suggesting that the O- and K-antigen encoding loci are subject to exceptionally high rates of recombination in isolates with the same genotype (Gavilan et al., 2013; Theethakaew et al., 2013). Herein, we agree that the high frequency of alterations in the O and/or K antigens is a significant biological characteristic of pandemic *V. parahaemolyticus* strains, which might be an important means of survival in the face of changing external environments and host immunological resistance.

Regional persistence of the clinical pandemic O3:K6 serotype has been identified in coastal areas of many countries, such as Mexico(2004–2015) (Velazquez-Roman et al., 2012; de Jesús Hernández-Díaz et al., 2015), Peru(2007) (Gil et al., 2007), Chile(2007–2009) (Cabello et al., 2007; Garcia et al., 2009), China(2007–2012) (Zhang et al., 2013; Li et al., 2014), India(2001–2012) (Pazhani et al., 2014), and Thailand(2006–2010) (Thongjun et al., 2013). However, it is not obvious what specific factors conferred upon this serotype the ability to disseminate around world. Some environmental conditions (e.g., seawater temperature, PH or salinity effects) affecting survival and the unique pathogenic potential of pandemic O3:K6 strains vs. other strains have been compared, but the specific advantage of pandemic O3:K6 strains over other strains remains unclear (Wong et al., 2000; Yeung et al., 2002). Further, investigations should focus on revealing the routes and mechanisms of the rapid spread of the pandemic clone.

In addition to the O3:K6 serotype, other pandemic serotypes have been isolated in both clinical and environmental samples from some certain countries, such as O1:KUT and O4:K48 in China (Chao et al., 2009), O1:K25 in Japan (Hara-Kudo et al., 2003) and O3:KUT, O10:KUT, and OUT:OUT in Mexico (Velazquez-Roman et al., 2012; de Jesús Hernández-Díaz et al., 2015). Although, the specific relationships between environmental serotypes and those leading to illnesses have not been determined, it is important to first understand epidemic situation of these serotypes through active surveillance.

In the present study, we showed that the population structure of *V. parahaemolyticus* was extremely genetically diverse based on the successful identification of 19 CCs and a large number of singletons, in agreement with previous findings (Han et al., 2015). Over half of the pandemic STs belonged to CC3 according to goeBURST analysis. The *dtdS* and *pntA* genes were found to be perfectly conserved throughout the evolution of CC3, whereas they presented some degree of polymorphism in pandemic strains. In our analysis, none of the values of Tajima's D was significantly different from zero (*P* > 0.10), suggesting that the housekeeping genes of the pandemic strains evolve under a random process (“neutrally”) and are subject to low selective pressure. The similar conclusion was obtained in studies based on the entire *V. parahaemolyticus* population (Theethakaew et al., 2013).

According to the available data, 64.3% of the STs (9/14) of the pandemic clones were isolated from China, suggesting that this country represents an important reservoir for the emergence of novel pandemic strains. If a global network for the prevention and control of *V. parahaemolyticus* infection is established in the future, the coastal regions of China should be recognized as important monitoring points. Three special STs (ST283, ST301, and ST302) typed in pandemic isolates originating from China were identified as singletons presenting distant relationships with other STs of the pandemic clone in this study. However, in a study by Chen et al. (2012), the corresponding strains were clustered together with other pandemic strains based on other molecular typing methods, such as enterobacterial repetitive intergenic consensus sequence PCR (ERIC-PCR) and sequence analysis of the gyrB gene. Thus, it can be observed that current molecular typing methods, including MLST, could lead to controversial results, making it difficult to draw conclusions, although such methods have been confirmed to provide a high level of resolution and information for elucidating the evolution of the *V. parahaemolyticus* clonal complex (Chen et al., 2012). Therefore, to accurately portray the relationships among strains at the molecular level, combined use of different molecular typing techniques with better discrimination could be considered in epidemiological investigations of *V. parahaemolyticus*. Whole genome sequencing (WGS), a powerful typing method with a robust differentiation ability for characterizing related isolates, is another outstanding alternative for analyzing the evolution and population structure of *V. parahaemolyticus* (Haendiges et al., 2015).

Invalid data in the pubMLST database were one problem restricting our analysis in this study. As of 15th July 2015, a total of 1844 records of isolates had been deposited, but definite STs were only available for 1700. Moreover, information on the corresponding biological characteristics of many uploaded isolates, such as sample sources, regions, drug sensitivity, serotypes and virulence genes was deficient. This lack of information is not conducive to conducting further epidemiologic and etiologic analyses of *V. parahaemolyticus* at a global scale. In this study, for five STs belonging to CC3 (ST557, ST787, ST886, ST1139, and ST1172), it could not be determined whether they were associated with pandemic clone, because of missing of toxRS/*new* gene and/or *tdh* gene sequences. As MLST assays play an important role in studies on the molecular epidemiology of *V. parahaemolyticus*, we recommend that the researchers uploaded their data on isolates as accurately and completely as possible.

In summary, the present study provides novel information on the abundance and prevalence of pandemic *V. parahaemolyticus* based on the analysis of clinical and environmental isolates from a worldwide collection. We showed that the regional persistence of pandemic O3:K6 has been established in coastal areas of many countries. The presence and persistence of pandemic *V. parahaemolyticus* strains, and especially the continuous appearance of environmental pandemic strains, is a matter of concern for public health authorities. We analyzed the genetic diversity of the pandemic clone to provide a comprehensive understanding of the microevolutionary relationships between pandemic strains. The answers to some unresolved questions about the pandemic clone, such as the advantage of pandemic O3:K6 over other strains and the mechanisms underlying the spread of strains with pandemic genetic marks, remain speculative, and require further investigations.

## Author contributions

Conceived and designed the experiments: DH, HT, CH. Performed the experiments: DH, CR. Analyzed the data: DH, HT, XZ. Contributed reagents/materials/analysis tools: DH, CH. Wrote the paper: DH.

### Conflict of interest statement

The authors declare that the research was conducted in the absence of any commercial or financial relationships that could be construed as a potential conflict of interest.
