# Supplementary material for: Sero-Prevalence and Genetic Diversity of Pandemic V. parahaemolyticus Strains Occurring at a Global Scale
Source: Front Microbiol. 2016 Apr 22;7:567. doi: 10.3389/fmicb.2016.00567 (PMC4840284; doi:10.3389/fmicb.2016.00567)
Supplement: Supplementary file 1 [file Table1.DOCX]

**Table S1. The 267 representative clinical and environmental *V. parahaemolyticus* isolates with pandemic genetic marks (*toxRS*/new+, *tdh*+ and *trh*-) collected from various countries and different times used in this study**

| **Id** | **Isolate** | **Country** | **Region** | **Year** | **Serotype** | **Source** | **Reference** |
| --- | --- | --- | --- | --- | --- | --- | --- |
| 1 | 220 | Angola | unknown | 1999 | O3:K6 | clinical | (Ellingsen et al., 2008) |
| 2 | AN-5034 | Bangladesh | unknown | 1998 | O4:K68 | clinical | (Gonzalez-Escalona et al., 2008) |
| 3 | S071_1247 | Bangladesh | unknown | 1998 | O1:KUT | clinical | (Han et al., 2008) |
| 4 | S072_1248 | Bangladesh | unknown | 1998 | O3:K6 | clinical | (Han et al., 2008) |
| 5 | AO-24491 | Bangladesh | unknown | 1999 | O1:K25 | clinical | (Nair et al., 2007) |
| 6 | AP-11243 | Bangladesh | unknown | 2000 | O1:KUT | clinical | (Gonzalez-Escalona et al., 2008) |
| 7 | unassigned | Bangladesh | Chittagong | 2001 | O3:K6 | aquatic environment | (Islam et al., 2004) |
| 8 | unassigned | Brazil | Ceara and Alagoas | 2002 | O3:K6 | clinical | (Velazquez-Roman et al., 2014) |
| 9 | unassigned | Brazil | Pernambuco and Alagoas | 2002 | O3:Kut | clinical | (Velazquez-Roman et al., 2014) |
| 10 | unassigned | Chile | unknown | 1998 | O3:K6 | clinical | (Velazquez-Roman et al., 2014) |
| 11 | PMA18.8 | Chile | Regio´n de Los Lagos | 2008 | O3:K6 | Sellfish | (Garcia et al., 2009) |
| 12 | unassigned | Chile | Puerto Montt | 2007 | O3:K6 | clinical | (Harth et al., 2009) |
| 13 | unassigned | Chile | Puerto Montt | 2007 | O3:K59 | clinical | (Harth et al., 2009) |
| 14 | unassigned | Chile | Puerto Montt | 2007 | O3:K6,59 | clinical | (Harth et al., 2009) |
| 15 | PMA109.5 | Chile | Puerto Montt | 2005 | O3:K6 | environmental | (Gonzalez-Escalona et al., 2008) |
| 16 | PMC-46 | Chile | Puerto Montt | 2004 | O4:K12 | clinical | (Nair et al., 2007) |
| 17 | unassigned | China | Shanghai | 2010-2012 | O3:K6 | clinical | (CHEN et al., 2014) |
| 18 | unassigned | China | Shanghai | 2010-2012 | O4:K68 | clinical | (CHEN et al., 2014) |
| 19 | unassigned | China | Shanghai | 2010-2012 | O1:K25 | clinical | (CHEN et al., 2014) |
| 20 | unassigned | China | Shanghai | 2010-2012 | O1:K36 | clinical | (CHEN et al., 2014) |
| 21 | unassigned | China | Shanghai | 2010-2012 | O1:KUT | clinical | (CHEN et al., 2014) |
| 22 | unassigned | China | Shanghai | 2010-2012 | O3:KUT | clinical | (CHEN et al., 2014) |
| 23 | unassigned | China | Shanghai | 2010-2012 | O5:KUT | clinical | (CHEN et al., 2014) |
| 24 | unassigned | China | Shanghai | 2010-2012 | O2:K3 | clinical | (CHEN et al., 2014) |
| 25 | unassigned | China | Shanghai | 2010-2012 | O10:K60 | clinical | (CHEN et al., 2014) |
| 26 | unassigned | China | Shanghai | 2010-2012 | O3:K3 | clinical | (CHEN et al., 2014) |
| 27 | SH11VP048 | China | Shanghai | 2011 | O3:K6 | clinical | pubMLST database |
| 28 | 82 | China | Beijing | 2010 | unknown | clinical | (Yan-yan et al., 2013) |
| 29 | unassigned | China | Shanghai | 2009-2011 | O3:K8 | clinical | (Li W et al., 2014) |
| 30 | SH2009017 | China | Shanghai | 2009 | O1:KUT | clinical | (Li W et al., 2014) |
| 31 | SH2011110 | China | Shanghai | 2011 | O4:K68 | clinical | (Li W et al., 2014) |
| 32 | SH2009009 | China | Shanghai | 2009 | O1:K36 | clinical | (Li W et al., 2014) |
| 33 | unassigned | China | Shenzhen | 2007-2012 | O3:K6 | clinical | (Li et al., 2014) |
| 34 | unassigned | China | Shenzhen | 2007-2012 | O1:KUT | clinical | (Li et al., 2014) |
| 35 | unassigned | China | Shenzhen | 2007-2012 | O1:K36 | clinical | (Li et al., 2014) |
| 36 | unassigned | China | Shenzhen | 2007-2012 | O4:K68 | clinical | (Li et al., 2014) |
| 37 | unassigned | China | Shenzhen | 2007-2012 | O5:K68 | clinical | (Li et al., 2014) |
| 38 | unassigned | China | Shenzhen | 2007-2012 | O1:K25 | clinical | (Li et al., 2014) |
| 39 | unassigned | China | Jiangsu | 2005-2008 | O4:K48 | Metapenaeus ensis | (Chao et al., 2009) |
| 40 | unassigned | China | Jiangsu | 2005-2008 | O3:K6 | Qingchuan fish | (Chao et al., 2009) |
| 41 | unassigned | China | Jiangsu | 2005-2008 | O3:K6 | Ribbon fish | (Chao et al., 2009) |
| 42 | unassigned | China | Jiangsu | 2005-2008 | O3:K6 | Crab | (Chao et al., 2009) |
| 43 | unassigned | China | Jiangsu | 2005-2008 | O1:KUT | environmental | (Chao et al., 2009) |
| 44 | unassigned | China | Jiangsu | 2005-2008 | O3:K6 | Metapenaeus ensis | (Chao et al., 2009) |
| 45 | unassigned | China | Jiangsu | 2005-2008 | O3:K6 | Salmon | (Chao et al., 2009) |
| 46 | unassigned | China | Jiangsu | 2005-2008 | O3:K6 | Thamnaconus septentrionalis | (Chao et al., 2009) |
| 47 | unassigned | China | Jiangsu | 2005-2008 | O3:K6 | Bombay duck | (Chao et al., 2009) |
| 48 | unassigned | China | Jiangsu | 2005-2008 | O3:K6 | Clam | (Chao et al., 2009) |
| 49 | unassigned | China | Jiangsu | 2005-2008 | O3:K6 | Seajelly | (Chao et al., 2009) |
| 50 | unassigned | China | Shanghai | 2005-2008 | O3:K6 | Pomfret | (Chao et al., 2009) |
| 51 | unassigned | China | Shanghai | 2005-2008 | O4:K68 | Pomfret | (Chao et al., 2009) |
| 52 | unassigned | China | Jiangsu | 2005-2008 | O1:KUT | clinical | (Chao et al., 2009) |
| 53 | V323 | China | Jiangsu | 2006 | O1:KUT | clinical | (Chao et al., 2009) |
| 54 | V275 | China | Jiangsu | 2006 | O1:K36 | clinical | (Chao et al., 2009) |
| 55 | V327 | China | Jiangsu | 2007 | O1:K26 | clinical | (Chao et al., 2009) |
| 56 | unassigned | China | Jiangsu | 2005-2008 | O1:K25 | clinical | (Chao et al., 2009) |
| 57 | V377 | China | Jiangsu | 2008 | O1:K56 | clinical | (Chao et al., 2009) |
| 58 | V504 | China | Jiangsu | 2009 | O3:K6 | clinical | (Chao et al., 2009) |
| 59 | V506 | China | Jiangsu | 2009 | O3:K6 | clinical | (Chao et al., 2009) |
| 60 | V283 | China | Jiangsu | 2007 | O3:K25 | clinical | (Chao et al., 2009) |
| 61 | V258 | China | Jiangsu | 2006 | O3:K68 | clinical | (Chao et al., 2009) |
| 62 | unassigned | China | Jiangsu | 2005-2008 | O4:K48 | clinical | (Chao et al., 2009) |
| 63 | unassigned | China | Jiangsu | 2005-2008 | O4:K68 | clinical | (Chao et al., 2009) |
| 64 | unassigned | China | Guangdong | 2008-2010 | O3:K6 | clinical | (Ma et al., 2011) |
| 65 | unassigned | China | Guangdong | 2008-2010 | O1:KUT | clinical | (Ma et al., 2011) |
| 66 | unassigned | China | Guangdong | 2008-2010 | O4:K8 | clinical | (Ma et al., 2011) |
| 67 | S063_1346 | China | Taiwan | 1998 | O1:K25 | clinical | (Han et al., 2008) |
| 68 | S064_1347 | China | Taiwan | 1998 | O3:K6 | clinical | (Han et al., 2008) |
| 69 | S065_1267 | China | Taiwan | 1998 | O1:K25 | clinical | (Han et al., 2008) |
| 70 | S066_1152 | China | Taiwan | 1997 | O3:K6 | clinical | (Han et al., 2008) |
| 71 | S067_1153 | China | Taiwan | 1997 | O3:K6 | clinical | (Han et al., 2008) |
| 72 | S068_1155 | China | Taiwan | 1997 | O3:K6 | clinical | (Han et al., 2008) |
| 73 | S074_1139 | China | Taiwan | 1997 | O3:K6 | clinical | (Han et al., 2008) |
| 74 | S075_1227 | China | Taiwan | 1999 | O3:K6 | clinical | (Han et al., 2008) |
| 75 | S077_1229 | China | Taiwan | 1997 | O3:K6 | clinical | (Han et al., 2008) |
| 76 | S090_1362 | China | Taiwan | 1999 | O4:K68 | clinical | (Han et al., 2008) |
| 77 | S095_1301 | China | Taiwan | 1996 | O3:K6 | clinical | (Han et al., 2008) |
| 78 | ICDC-VP77 | China | Guangxi | 2003 | O3:K6 | clinical | (Han et al., 2008) |
| 79 | ICDC-VP87 | China | Guangxi | 2004 | O3:K6 | clinical | (Han et al., 2008) |
| 80 | ICDC-VP88 | China | Guangxi | 2005 | O3:K6 | clinical | (Han et al., 2008) |
| 81 | ICDC-VP133 | China | Hebei | 2007 | O3:K6 | clinical | (Han et al., 2008) |
| 82 | ICDC-VP53 | China | Liaoning | 2005 | O3:K6 | clinical | (Han et al., 2008) |
| 83 | ZJ3 | China | Zhejiang | 2003 | O1:KUT | clinical | (Vongxay et al., 2008) |
| 84 | ZJ17 | China | Zhejiang | 2003 | O3:K6 | clinical | (Vongxay et al., 2008) |
| 85 | HZ 34 | China | Zhejiang | 2004 | O3:K6 | Shellfish | (Vongxay et al., 2008) |
| 86 | BA2 | China | Shenzhen | 2008 | O3:K6 | clinical | (Ju et al., 2015) |
| 87 | GM6 | China | Shenzhen | 2008 | O4:K68 | clinical | (Ju et al., 2015) |
| 88 | W1 | China | Shenzhen | 2006 | O1:K25 | clinical | (Ju et al., 2015) |
| 89 | W50 | China | Shenzhen | 2007 | O11:K36 | clinical | (Ju et al., 2015) |
| 90 | 07VP518 | China | Jiangsu | 2007 | O3:K29 | clinical | (Li W et al., 2014) |
| 91 | 09VP157 | China | Zhejiang | 2009 | O3:K6 | clinical | (Li W et al., 2014) |
| 92 | 07VP521 | China | Jiangsu | 2007 | O1:K25 | clinical | (Li W et al., 2014) |
| 93 | 09VP45 | China | Sichuan | 2009 | O3:K6 | clinical | (Li W et al., 2014) |
| 94 | 07VP541 | China | Jiangsu | 2009 | O3:K6 | clinical | (Li W et al., 2014) |
| 95 | 07VP155 | China | Guangxi | 2007 | O3:K6 | clinical | (Li W et al., 2014) |
| 96 | 09VP170 | China | Zhejiang | 2009 | O1:K36 | clinical | (Li W et al., 2014) |
| 97 | 07VP531 | China | Jiangsu | 2007 | O1:K5 | clinical | (Li W et al., 2014) |
| 98 | 09VP167 | China | Zhejiang | 2009 | O1:K5 | clinical | (Li W et al., 2014) |
| 99 | F6 | China | shanghai | 2006 | O1:Kut | Ribbon fish | (Chen et al., 2012) |
| 100 | F13 | China | zhoushan | 2007 | O3:K6 | Ribbon fish | (Chen et al., 2012) |
| 101 | P15 | China | shanghai | 2006 | O4:KUT | clinical | (Chen et al., 2012) |
| 102 | P7 | China | shanghai | 2006 | O3:K6 | clinical | (Chen et al., 2012) |
| 103 | P32 | China | shanghai | 2006 | O4:K8 | clinical | (Chen et al., 2012) |
| 104 | P39 | China | shanghai | 2006 | O4:K68 | clinical | (Chen et al., 2012) |
| 105 | P43 | China | shanghai | 2007 | O4:KUT | clinical | (Chen et al., 2012) |
| 106 | P75 | China | shanghai | 2007 | Out:Kut | clinical | (Chen et al., 2012) |
| 107 | P86 | China | shanghai | 2007 | O1:KUT | clinical | (Chen et al., 2012) |
| 108 | P202 | China | shanghai | 2007 | O4:K8 | clinical | (Chen et al., 2012) |
| 109 | P196 | China | shanghai | 2007 | O4:K68 | clinical | (Chen et al., 2012) |
| 110 | QD2 | China | Qingdao | 2006 | O3:K6 | clinical | (Chen et al., 2012) |
| 111 | NB24 | China | Ningbo | 2008 | O1:KUT | clinical | (Chen et al., 2012) |
| 112 | NB755 | China | Ningbo | 2006 | O4:KUT | clinical | (Chen et al., 2012) |
| 113 | NB40 | China | Ningbo | 2008 | O3:K6 | clinical | (Chen et al., 2012) |
| 114 | unassigned | China | Taiwan | 2005 | O6:K18 | clinical | (Nair et al., 2007) |
| 115 | SH11VP48 | China | shanghai | 2011 | O3:K6 | clinical | pubMLST database |
| 116 | Hangzhou2010-599 | China | Zhejiang | 2010 | O3:K6 | clinical | pubMLST database |
| 117 | 222 | Ecuador | unknown | 1999 | O3:K6 | clinical | (Ellingsen et al., 2008) |
| 118 | unassigned | France | unknown | 2004 | O3:K6 | clinical | (Quilici et al., 2005) |
| 119 | 970136 | France | Atlantic coast | 1997 | O3:K6 | oysters | (Quilici et al., 2005) |
| 120 | 980402 | France | Southwest | 1998 | O3:K6 | shellfish | (Quilici et al., 2005) |
| 121 | 990346 | France | Mediterranean coast | 1999 | O3:K6 | unknown | (Quilici et al., 2005) |
| 122 | IDH525 | India | Kolkata | 2011 | O1:K30 | clinical | (Pazhani et al., 2014) |
| 123 | VPHY145 | India | Kolkata | 2001 | O1:K38 | clinical | (Pazhani et al., 2014) |
| 124 | IDH3722 | India | Kolkata | 2011 | O1:K56 | clinical | (Pazhani et al., 2014) |
| 125 | L11159 | India | Kolkata | 2005 | O2:K4 | clinical | (Pazhani et al., 2014) |
| 126 | IDH1580 | India | Kolkata | 2009 | O4:KUT | clinical | (Pazhani et al., 2014) |
| 127 | unassigned | India | Kolkata | unknown | O4:K4 | clinical | (Pazhani et al., 2014) |
| 128 | IDH2921 | India | Kolkata | 2010 | O4:K13 | clinical | (Pazhani et al., 2014) |
| 129 | L15489 | India | Kolkata | 2005 | O4:K25 | clinical | (Pazhani et al., 2014) |
| 130 | G12210 | India | Kolkata | 2001 | O4:K55 | clinical | (Pazhani et al., 2014) |
| 131 | L3834 | India | Kolkata | 2006 | O8:K21 | clinical | (Pazhani et al., 2014) |
| 132 | IDH4796 | India | Kolkata | 2012 | O10:K60 | clinical | (Pazhani et al., 2014) |
| 133 | G9652 | India | Kolkata | 2001 | O1:KUT | clinical | (Pazhani et al., 2014) |
| 134 | G10956 | India | Kolkata | 2004 | O1:K25 | clinical | (Pazhani et al., 2014) |
| 135 | K4202 | India | Kolkata | 2005 | O1:K25 | clinical | (Pazhani et al., 2014) |
| 136 | IDH1576 | India | Kolkata | 2009 | O1:K25 | clinical | (Pazhani et al., 2014) |
| 137 | IDH2916 | India | Kolkata | 2010 | O1:K25 | clinical | (Pazhani et al., 2014) |
| 138 | ATCC BAA-241 | India | Kolkata | 1998 | O4:K68 | clinical | (Ansaruzzaman et al., 2008) |
| 139 | S091_1377 | India | unknown | 1999 | O4:K68 | clinical | (Han et al., 2008) |
| 140 | ATCC BAA-242 | India | unknown | 1998 | O1:KUT | clinical | (Nair et al., 2007) |
| 141 | VP81 | India | Kolkata | 1996 | O3:K6 | clinical | (Nair et al., 2007) |
| 142 | unassigned | India | unknown | 2004 | O5:Kut | clinical | (Nair et al., 2007) |
| 143 | unassigned | India | unknown | 2002 | O5:K17 | clinical | (Nair et al., 2007) |
| 144 | unassigned | India | unknown | 2002 | O5:K25 | clinical | (Nair et al., 2007) |
| 145 | unassigned | India | unknown | 2002 | O1:K33 | clinical | (Nair et al., 2007) |
| 146 | unassigned | India | unknown | 2002 | O2:K3 | clinical | (Nair et al., 2007) |
| 147 | unassigned | India | unknown | 2003-2004 | OUT:KUT | clinical | (Nair et al., 2007) |
| 148 | unassigned | India | unknown | 2003-2004 | O3:KUT | clinical | (Nair et al., 2007) |
| 149 | unassigned | India | unknown | 2004 | O3:K5 | clinical | (Nair et al., 2007) |
| 150 | unassigned | India | unknown | 2004 | O4:K4 | clinical | (Nair et al., 2007) |
| 151 | unassigned | India | unknown | 2004 | O4:K10 | clinical | (Nair et al., 2007) |
| 152 | unassigned | India | southwest coast | 2002 | O3:K6 | Oysters | (Deepanjali et al., 2005) |
| 153 | S079_1221 | Indonesia | unknown | 1997 | O3:K6 | clinical | (Han et al., 2008) |
| 154 | CEREM 38845 | Italy | Central Italy | 2008 | O3:K6 | clinical | (Ottaviani et al., 2010) |
| 155 | unassigned | Italy | Northern Italy | 2007 | O3:K6 | Seawater and Plankton samples | (Ju et al., 2015) |
| 156 | unassigned | Italy | central Italy | 2007 | O3:K6 | clinical | (Ottaviani et al., 2008) |
| 157 | unassigned | Japan | Saitama City | 2013 | O10:K60 | clinical | (Ueno et al., 2015) |
| 158 | S083_1249 | Japan | unknown | 1998 | O3:K6 | clinical | (Han et al., 2008) |
| 159 | VPKX (RIMD 2210633) | Japan | unknown | 1996 | O3:K6 | clinical | (Gonzalez-Escalona et al., 2008) |
| 160 | VP1152 | Japan | north Japan | 2001 | O3:K6 | Rock oyster | (Hara-Kudo et al., 2003) |
| 161 | VPF01-13 | Japan | central Japan | 2001 | O3:K6 | Hen clam | (Hara-Kudo et al., 2003) |
| 162 | VPF01-5 | Japan | central Japan | 2001 | O3:K6 | Short-neck clam | (Hara-Kudo et al., 2003) |
| 163 | VPF00-18 | Japan | central Japan | 2000 | O3:K6 | Short-neck clam | (Hara-Kudo et al., 2003) |
| 164 | APCC VP 00157 | Japan | north Japan | 2001 | O1:K25 | Sediment of river | (Hara-Kudo et al., 2003) |
| 165 | APCC VP 00190 | Japan | north Japan | 2001 | O4:K68 | Sediment of river | (Hara-Kudo et al., 2003) |
| 166 | APCC VP 9810 | Japan | north Japan | 1998 | O3:K6 | Sediment of river | (Hara-Kudo et al., 2003) |
| 167 | KXV-641 | Japan | unknown | 1998 | O1:K25 | clinical | (Gonzalez-Escalona et al., 2008) |
| 168 | S081_1203 | Korea | unknown | 1997-1998 | O3:K6 | clinical | (Han et al., 2008) |
| 169 | VP2 | Korea | unknown | 1998 | O3:K6 | clinical | (Gonzalez-Escalona et al., 2008) |
| 170 | 97LVP2 | Laos | unknown | 1997 | O3:K6 | clinical | (Matsumoto et al., 2000) |
| 171 | unassigned | Mexico | Northwest Mexico | 2004-2010 | O3:Kut | clinical | (Velazquez-Roman et al., 2012) |
| 172 | unassigned | Mexico | Pacific coast of Sinaloa | 2004-2010 | O3:K6 | Sellfish | (Velazquez-Roman et al., 2012) |
| 173 | unassigned | Mexico | Northwest Mexico | 2004-2010 | O4:K12 | clinical | (Velazquez-Roman et al., 2012) |
| 174 | unassigned | Mexico | Pacific coast of Sinaloa | 2004-2010 | O10:KUT | Sediment | (Velazquez-Roman et al., 2012) |
| 175 | unassigned | Mexico | Pacific coast of Sinaloa | 2004-2010 | OUT:KUT | Seawater | (Velazquez-Roman et al., 2012) |
| 176 | unassigned | Mexico | Pacific coast of Sinaloa | 2011-2013 | O3:K6 | clinical | (de JesÃºs HernÃ Ndez-DÃ Az et al., 2015) |
| 177 | unassigned | Mexico | Pacific coast of Sinaloa | 2011-2013 | O3:K29 | clinical | (de JesÃºs HernÃ Ndez-DÃ Az et al., 2015) |
| 178 | unassigned | Mexico | Pacific coast of Sinaloa | 2011-2013 | OUT:KUT | clinical | (de JesÃºs HernÃ Ndez-DÃ Az et al., 2015) |
| 179 | unassigned | Mexico | Pacific coast of Sinaloa | 2011-2013 | O3:K6 | Shrimp | (de JesÃºs HernÃ Ndez-DÃ Az et al., 2015) |
| 180 | unassigned | Mexico | Pacific coast of Sinaloa | 2011-2013 | O3:KUT | Shrimp and Seawater | (de JesÃºs HernÃ Ndez-DÃ Az et al., 2015) |
| 181 | unassigned | Mexico | Pacific coast of Sinaloa | 2011-2013 | O3:KUT | Sediment | (de JesÃºs HernÃ Ndez-DÃ Az et al., 2015) |
| 182 | unassigned | Mexico | Sinaloa | 2012 | O4:K10 | olive ridley turtles | (Zavala-Norzagaray et al., 2015) |
| 183 | IB3892 | Mozambique | beria | 2004 | O3:K6 | clinical | (Ansaruzzaman et al., 2008) |
| 184 | IB3887 | Mozambique | beria | 2004 | O4:K68 | clinical | (Ansaruzzaman et al., 2008) |
| 185 | 227 | Norway | unknown | 2002 | O5:K68 | clinical | (Ellingsen et al., 2008) |
| 186 | 056-01 | Peru | unknown | 2001 | O3:K6 | clinical | (Gavilan et al., 2013) |
| 187 | 004-02 | Peru | unknown | 2002 | O3:K6 | clinical | (Gavilan et al., 2013) |
| 188 | 038-03 | Peru | unknown | 2003 | O3:K6 | clinical | (Gavilan et al., 2013) |
| 189 | 205-05 | Peru | unknown | 2005 | O1:KUT | clinical | (Gavilan et al., 2013) |
| 190 | 275-99 | Peru | unknown | 1999 | O3:K58 | clinical | (Gavilan et al., 2013) |
| 191 | 276-99 | Peru | unknown | 1999 | O3:K6 | clinical | (Gavilan et al., 2013) |
| 192 | 304-07 | Peru | unknown | 2007 | O3:K30 | clinical | (Gavilan et al., 2013) |
| 193 | 330-00 | Peru | unknown | 2000 | O3:K6 | clinical | (Gavilan et al., 2013) |
| 194 | 784-98 | Peru | unknown | 1998 | O3:K6 | clinical | (Gavilan et al., 2013) |
| 195 | 369-07 | Peru | unknown | 2007 | O3:Kunk | clinical | (Gavilan et al., 2013) |
| 196 | 301-07 | Peru | unknown | 2007 | O1:KUT | clinical | (Gavilan et al., 2013) |
| 197 | 906-97 | Peru | unknown | 1997 | O3:K6 | clinical | (Gavilan et al., 2013) |
| 198 | unassigned | Peru | unknown | 1998 | O3:K68 | clinical | (Gil et al., 2007) |
| 199 | unassigned | Peru | unknown | 1998 | O3:K58 | clinical | (Gil et al., 2007) |
| 200 | unassigned | Peru | unknown | 1998 | OUT:K6 | clinical | (Gil et al., 2007) |
| 201 | unassigned | Russia | Primorsky Region | 2012 | O3:K6 | clinical | (Rykovskaia et al., 2014) |
| 202 | S061_1262 | Singapore | unknown | 1998 | O3:K6 | clinical | (Han et al., 2008) |
| 203 | S062_1263 | Singapore | unknown | 1998 | O6:K18 | clinical | (Han et al., 2008) |
| 204 | S084_1172 | Singapore | unknown | 1996 | O3:K6 | clinical | (Han et al., 2008) |
| 205 | S087_1264 | Singapore | unknown | 1998 | O4:K68 | clinical | (Han et al., 2008) |
| 206 | 2888339 | Spain | A Coruña | 2004 | O3:K6 | clinical | (Martinez-Urtaza et al., 2005) |
| 207 | unassigned | Spain | A Coruña | 2004 | O3:Kut | clinical | (Martinez-Urtaza et al., 2005) |
| 208 | unassigned | Thailand | Songkhla | 2006-2010 | O3:K6 | clinical | (Thongjun et al., 2013) |
| 209 | unassigned | Thailand | Songkhla | 2006-2010 | O4:K8 | clinical | (Thongjun et al., 2013) |
| 210 | unassigned | Thailand | Songkhla | 2006-2010 | O1:K25 | clinical | (Thongjun et al., 2013) |
| 211 | unassigned | Thailand | Songkhla | 2006-2010 | O1:KUT | clinical | (Thongjun et al., 2013) |
| 212 | unassigned | Thailand | Songkhla | 2006-2010 | O4:K9 | clinical | (Thongjun et al., 2013) |
| 213 | unassigned | Thailand | Songkhla | 2006-2010 | O3:KUT | clinical | (Thongjun et al., 2013) |
| 214 | unassigned | Thailand | Songkhla | 2000 | O3:K6 | clinical | (Wootipoom et al., 2007) |
| 215 | VPHY145 | Thailand | Songkhla | 1999 | O4:K68 | clinical | (Gonzalez-Escalona et al., 2008) |
| 216 | unassigned | Thailand | Songkhla | 2000 | O4:K68 | clinical | (Wootipoom et al., 2007) |
| 217 | unassigned | Thailand | Songkhla | 2000 | O1:K25 | clinical | (Wootipoom et al., 2007) |
| 218 | unassigned | Thailand | Songkhla | 2000 | O2:K3 | clinical | (Wootipoom et al., 2007) |
| 219 | unassigned | Thailand | Songkhla | 2001 | O3:K6 | clinical | (Wootipoom et al., 2007) |
| 220 | unassigned | Thailand | Songkhla | 2001 | O4:K68 | clinical | (Wootipoom et al., 2007) |
| 221 | unassigned | Thailand | Songkhla | 2001 | O1:K25 | clinical | (Wootipoom et al., 2007) |
| 222 | unassigned | Thailand | Songkhla | 2001 | O1:Kut | clinical | (Wootipoom et al., 2007) |
| 223 | unassigned | Thailand | Songkhla | 2002 | O3:K6 | clinical | (Wootipoom et al., 2007) |
| 224 | unassigned | Thailand | Songkhla | 2002 | O3:K29 | clinical | (Wootipoom et al., 2007) |
| 225 | unassigned | Thailand | Songkhla | 2002 | O1:K25 | clinical | (Wootipoom et al., 2007) |
| 226 | unassigned | Thailand | Songkhla | 2002 | O1:Kut | clinical | (Wootipoom et al., 2007) |
| 227 | unassigned | Thailand | Songkhla | 2002 | O1:K41 | clinical | (Wootipoom et al., 2007) |
| 228 | unassigned | Thailand | Songkhla | 2003 | O3:K6 | clinical | (Wootipoom et al., 2007) |
| 229 | unassigned | Thailand | Songkhla | 2003 | O3:K29 | clinical | (Wootipoom et al., 2007) |
| 230 | unassigned | Thailand | Songkhla | 2003 | O1:K25 | clinical | (Wootipoom et al., 2007) |
| 231 | unassigned | Thailand | Songkhla | 2003 | O1:Kut | clinical | (Wootipoom et al., 2007) |
| 232 | unassigned | Thailand | Songkhla | 2003 | O5:Kut | clinical | (Wootipoom et al., 2007) |
| 233 | unassigned | Thailand | Songkhla | 2003 | O4:K68 | clinical | (Wootipoom et al., 2007) |
| 234 | unassigned | Thailand | Songkhla | 2003 | R:KUT | clinical | (Wootipoom et al., 2007) |
| 235 | unassigned | Thailand | Songkhla | 2004 | O3:K6 | clinical | (Wootipoom et al., 2007) |
| 236 | unassigned | Thailand | Songkhla | 2004 | O1:K25 | clinical | (Wootipoom et al., 2007) |
| 237 | unassigned | Thailand | Songkhla | 2004 | O4:K68 | clinical | (Wootipoom et al., 2007) |
| 238 | unassigned | Thailand | Songkhla | 2004 | O1:Kut | clinical | (Wootipoom et al., 2007) |
| 239 | unassigned | Thailand | Songkhla | 2004 | O3:Kut | clinical | (Wootipoom et al., 2007) |
| 240 | unassigned | Thailand | Songkhla | 2004 | O3:K46 | clinical | (Wootipoom et al., 2007) |
| 241 | unassigned | Thailand | Songkhla | 2005 | O3:K6 | clinical | (Wootipoom et al., 2007) |
| 242 | unassigned | Thailand | Songkhla | 1999 | O1:K25 | clinical | (Laohaprertthisan et al., 2003) |
| 243 | unassigned | Thailand | Songkhla | 2005 | O1:K25 | clinical | (Wootipoom et al., 2007) |
| 244 | unassigned | Thailand | Songkhla | 2005 | O4:K68 | clinical | (Wootipoom et al., 2007) |
| 245 | unassigned | Thailand | Songkhla | 2005 | O1:Kut | clinical | (Wootipoom et al., 2007) |
| 246 | unassigned | Thailand | Songkhla | 2005 | O4:K4 | clinical | (Wootipoom et al., 2007) |
| 247 | S073_1023 | Thailand | unknown | 1997 | O3:K6 | clinical | (Han et al., 2008) |
| 248 | S094_1456 | Thailand | unknown | 1996 | O3:K6 | clinical | (Han et al., 2008) |
| 249 | VPHY191 | Thailand | unknown | 1999 | O1:K25 | clinical | (Han et al., 2008) |
| 250 | unassigned | Thailand | Songkhla | 1998–1999 | O4:K12 | clinical | (Laohaprertthisan et al., 2003) |
| 251 | KX-V641 | Thailand | Songkhla | 1998–1999 | O1:K25 | clinical | (Laohaprertthisan et al., 2003) |
| 252 | unassigned | Thailand | Songkhla | 1998-1999 | O3:K6 | shellﬁsh | (Vuddhakul et al., 2000) |
| 253 | VPT 82 | Thailand | Songkhla | 2001 | O3:K46 | clinical | (Wootipoom et al., 2007) |
| 254 | KX-V829 | Thailand | Songkhla | 1998–1999 | O1:K41 | clinical | (Laohaprertthisan et al., 2003) |
| 255 | unassigned | UK | unknown | 2012 | O3:K6 | Sellfish product | (Powell et al., 2013) |
| 256 | VP17MD | USA | Marylaunknown | 2012 | O3:K6 | clinical | pubMLST database |
| 257 | BAC-98-3374 | USA | unknown | 1998 | O3:K6 | clinical | (Gonzalez-Escalona et al., 2008) |
| 258 | unassigned | Vietnam | Khanh Hoa | 1998–1999 | O1:K25 | clinical | (Chowdhury et al., 2004) |
| 259 | unassigned | Vietnam | Khanh Hoa | 1998 | O4:K68 | clinical | (Chowdhury et al., 2004) |
| 260 | unassigned | Vietnam | Khanh Hoa | 1998–1999 | O1:K56 | clinical | (Nair et al., 2007) |
| 261 | unassigned | Vietnam | Khanh Hoa | 1998–1999 | O3:K75 | clinical | (Nair et al., 2007) |
| 262 | unassigned | Vietnam | Khanh Hoa | 1998–1999 | O4:K8 | clinical | (Nair et al., 2007) |
| 263 | unassigned | Vietnam | Khanh Hoa | 1998–1999 | O4:KUT | clinical | (Nair et al., 2007) |
| 264 | unassigned | Vietnam | Khanh Hoa | 1998–1999 | O5:Kut | clinical | (Nair et al., 2007) |
| 265 | unassigned | Vietnam | unknown | 1998–1999 | O4:K12 | clinical | (Nair et al., 2007) |
| 266 | unassigned | Vietnam | Khanh Hoa | 1998–1999 | O1:K41 | clinical | (Nair et al., 2007) |
| 267 | unassigned | Wietnam | Khanh Hoa | 1997 | O3:K6 | clinical | (Chowdhury et al., 2004) |

**REFERENCES:**

CHEN Hong-you,SHENG Yue-ying,SONG Yuan-jun,TU Li-hong,ZHANG Xi and CHEN Min.Serotypes and molecular characteristics of Vibrio parahaemolyticus pandemic strains in Shanghai[J]. Chin J Food Hygi,2014,26(1):5-9. (In Chinese)

Ansaruzzaman, M., Chowdhury, A., Bhuiyan, N.A., Sultana, M., Safa, A., and Lucas, M. (2008). Characteristics of a pandemic clone of O3 : K6 and O4 : K68 Vibrio parahaemolyticus isolated in Beira, Mozambique. *J. Med. Microbiol.* 57, 1502-7. doi: 10.1099/jmm.0.2008/004275-0.

Chao, G., Jiao, X., Zhou, X., Yang, Z., Huang, J., and Pan, Z. (2009). Serodiversity, pandemic O3:K6 clone, molecular typing, and antibiotic susceptibility of foodborne and clinical Vibrio parahaemolyticus isolates in Jiangsu, China. *Foodborne Pathog. Dis.* 6, 1021-8. doi: 10.1089/fpd.2009.0295.

Chen, W., Xie, Y., Xu, J., Wang, Q., Gu, M., and Yang, J. (2012). Molecular typing of Vibrio parahaemolyticus isolates from the middle-east coastline of China. *Int. J. Food Microbiol.* 153, 402-12. doi: 10.1016/j.ijfoodmicro.2011.12.001.

Chowdhury, A., Ishibashi, M., Thiem, V.D., Tuyet, D.T., Tung, T.V., and Chien, B.T. (2004). Emergence and serovar transition of Vibrio parahaemolyticus pandemic strains isolated during a diarrhea outbreak in Vietnam between 1997 and 1999. *Microbiol. Immunol.* 48, 319-27.

de JesÃºs HernÃ Ndez-DÃ Az, L., Leon-Sicairos, N., Velazquez-Roman, J., Flores-VillaseÃ Or, H.C., Guadron-Llanos, A.M., and Martinez-Garcia, J.J. (2015). A pandemic Vibrio parahaemolyticus O3:K6 clone causing most associated diarrhea cases in the Pacific Northwest coast of Mexico. *Front. Microbiol.* 6, 221. doi: 10.3389/fmicb.2015.00221.

Deepanjali, A., Kumar, H.S., Karunasagar, I., and Karunasagar, I. (2005). Seasonal variation in abundance of total and pathogenic Vibrio parahaemolyticus bacteria in oysters along the southwest coast of India. *Appl Environ Microbiol* 71, 3575-80. doi: 10.1128/AEM.71.7.3575-3580.2005.

Ellingsen, A.B., Jorgensen, H., Wagley, S., Monshaugen, M., and Rorvik, L.M. (2008). Genetic diversity among Norwegian Vibrio parahaemolyticus. *J. Appl. Microbiol.* 105, 2195-202. doi: 10.1111/j.1365-2672.2008.03964.x.

Garcia, K., Torres, R., Uribe, P., Hernandez, C., Rioseco, M.L., and Romero, J. (2009). Dynamics of clinical and environmental Vibrio parahaemolyticus strains during seafood-related summer diarrhea outbreaks in southern Chile. *Appl Environ Microbiol* 75, 7482-7. doi: 10.1128/AEM.01662-09.

Gavilan, R.G., Zamudio, M.L., and Martinez-Urtaza, J. (2013). Molecular epidemiology and genetic variation of pathogenic Vibrio parahaemolyticus in Peru. *PLoS Negl Trop Dis* 7, e2210. doi: 10.1371/journal.pntd.0002210.

Gil, A.I., Miranda, H., Lanata, C.F., Prada, A., Hall, E.R., and Barreno, C.M. (2007). O3:K6 serotype of Vibrio parahaemolyticus identical to the global pandemic clone associated with diarrhea in Peru. *Int. J. Infect. Dis.* 11, 324-8. doi: 10.1016/j.ijid.2006.08.003.

Gonzalez-Escalona, N., Martinez-Urtaza, J., Romero, J., Espejo, R.T., Jaykus, L.A., and DePaola, A. (2008). Determination of molecular phylogenetics of Vibrio parahaemolyticus strains by multilocus sequence typing. *J. Bacteriol.* 190, 2831-40. doi: 10.1128/JB.01808-07.

Han, H., Wong, H.C., Kan, B., Guo, Z., Zeng, X., and Yin, S. (2008). Genome plasticity of Vibrio parahaemolyticus: microevolution of the 'pandemic group'. *BMC Genomics* 9, 570. doi: 10.1186/1471-2164-9-570.

Hara-Kudo, Y., Sugiyama, K., Nishibuchi, M., Chowdhury, A., Yatsuyanagi, J., and Ohtomo, Y. (2003). Prevalence of pandemic thermostable direct hemolysin-producing Vibrio parahaemolyticus O3:K6 in seafood and the coastal environment in Japan. *Appl Environ Microbiol* 69, 3883-91.

Harth, E., Matsuda, L., Hernandez, C., Rioseco, M.L., Romero, J., and Gonzalez-Escalona, N. (2009). Epidemiology of Vibrio parahaemolyticus outbreaks, southern Chile. *Emerg. Infect. Dis.* 15, 163-8.

Islam, M.S., Tasmin, R., Khan, S.I., Bakht, H.B., Mahmood, Z.H., and Rahman, M.Z. (2004). Pandemic strains of O3:K6 Vibrio parahaemolyticus in the aquatic environment of Bangladesh. *Can. J. Microbiol.* 50, 827-34. doi: 10.1139/w04-072.

Ju, C., Yu, M., Huang, R., Luo, J., and Duan, Y. (2015). Genetic characterization of Vibrio parahaemolyticus O3: K6 serovariant isolated in Shenzhen. *Zhonghua Yu Fang Yi Xue Za Zhi* 49, 21-5.

Laohaprertthisan, V., Chowdhury, A., Kongmuang, U., Kalnauwakul, S., Ishibashi, M., and Matsumoto, C. (2003). Prevalence and serodiversity of the pandemic clone among the clinical strains of Vibrio parahaemolyticus isolated in southern Thailand. *Epidemiol. Infect.* 130, 395-406.

Li W, Mei L, Tang Z, Yang X, Li X and Pei X., et al. (2014). Analysis of molecular features of clinical Vibrio parahaemolyticus strains in China. *Chinese Journal of Preventive Medicine* 48, 44-52. (In Chinese). doi: 10.3760/cma.j.issn.0253-9624.2014.01.010.

Li, Y., Xie, X., Shi, X., Lin, Y., Qiu, Y., and Mou, J. (2014). Vibrio parahaemolyticus, Southern Coastal Region of China, 2007-2012. *Emerg. Infect. Dis.* 20, 685-8. doi: 10.3201/eid2004.130744.

Martinez-Urtaza, J., Simental, L., Velasco, D., DePaola, A., Ishibashi, M., and Nakaguchi, Y. (2005). Pandemic Vibrio parahaemolyticus O3:K6, Europe. *Emerg. Infect. Dis.* 11, 1319-20. doi: 10.3201/eid1108.050322.

Matsumoto, C., Okuda, J., Ishibashi, M., Iwanaga, M., Garg, P., and Rammamurthy, T. (2000). Pandemic spread of an O3:K6 clone of Vibrio parahaemolyticus and emergence of related strains evidenced by arbitrarily primed PCR and toxRS sequence analyses. *J. Clin. Microbiol.* 38, 578-85.

[Ma C](http://www.ncbi.nlm.nih.gov/pubmed/?term=Ma%20C%5BAuthor%5D&cauthor=true&cauthor_uid=24138080), [He D](http://www.ncbi.nlm.nih.gov/pubmed/?term=He%20D%5BAuthor%5D&cauthor=true&cauthor_uid=24138080), [Deng X](http://www.ncbi.nlm.nih.gov/pubmed/?term=Deng%20X%5BAuthor%5D&cauthor=true&cauthor_uid=24138080), [Ke B](http://www.ncbi.nlm.nih.gov/pubmed/?term=Ke%20B%5BAuthor%5D&cauthor=true&cauthor_uid=24138080),[Li B](http://www.ncbi.nlm.nih.gov/pubmed/?term=Li%20B%5BAuthor%5D&cauthor=true&cauthor_uid=24138080) and [Li W](http://www.ncbi.nlm.nih.gov/pubmed/?term=Li%20W%5BAuthor%5D&cauthor=true&cauthor_uid=24138080). (2011). Molecular characteristic of dominant serotypes of Vibrio parahaemolyticus isolated from foodborne disease outbreaks in Guangdong province. *Chinese Journal of Microbiology and Immunology.*31,1093-8. (In Chinese). doi: 10.3760/cma.j.issn.0254-5101.2011.12.009.

Nair, G.B., Ramamurthy, T., Bhattacharya, S.K., Dutta, B., Takeda, Y., and Sack, D.A. (2007). Global dissemination of Vibrio parahaemolyticus serotype O3:K6 and its serovariants. *Clin. Microbiol. Rev.* 20, 39-48. doi: 10.1128/CMR.00025-06.

Ottaviani, D., Leoni, F., Rocchegiani, E., Canonico, C., Potenziani, S., and Santarelli, S. (2010). Vibrio parahaemolyticus-associated gastroenteritis in Italy: persistent occurrence of O3:K6 pandemic clone and emergence of O1:KUT serotype. *Diagn Microbiol Infect Dis* 66, 452-5. doi: 10.1016/j.diagmicrobio.2009.11.015.

Ottaviani, D., Leoni, F., Rocchegiani, E., Santarelli, S., Canonico, C., and Masini, L. (2008). First clinical report of pandemic Vibrio parahaemolyticus O3:K6 infection in Italy. *J. Clin. Microbiol.* 46, 2144-5. doi: 10.1128/JCM.00683-08.

Pazhani, G.P., Bhowmik, S.K., Ghosh, S., Guin, S., Dutta, S., and Rajendran, K. (2014). Trends in the Epidemiology of Pandemic and Non-pandemic Strains of Vibrio parahaemolyticus Isolated from Diarrheal Patients in Kolkata, India. *PLoS Neglect. Trop. D.* 8, e2815. doi: 10.1371/journal.pntd.0002815.

Powell, A., Baker-Austin, C., Wagley, S., Bayley, A., and Hartnell, R. (2013). Isolation of Pandemic Vibrio parahaemolyticus from UK Water and Shellfish Produce. *Microb. Ecol.* 65, 924-927. doi: 10.1007/s00248-013-0201-8.

Quilici, M.L., Robert-Pillot, A., Picart, J., and Fournier, J.M. (2005). Pandemic Vibrio parahaemolyticus O3:K6 spread, France. *Emerg. Infect. Dis.* 11, 1148-9. doi: 10.3201/eid1107.041008.

Rykovskaia, O.A., Mazrukho, A.V., Smolikova, L.M., Monakhova, E.V., Chemisova, O.S., and Podoinitsyna, O.A. (2014). O3:K6 serogroup vibrio parahaemolyticus - the causative agent of food toxic infection outbreaks in Primorsky region of Russian federation. *Zh Mikrobiol Epidemiol Immunobiol*, 57-61. doi.

Thongjun, J., Mittraparp-arthorn, P., Yingkajorn, M., Kongreung, J., Nishibuchi, M., and Vuddhakul, V. (2013). The Trend of Vibrio parahaemolyticus Infections in Southern Thailand from 2006 to 2010. *Tropical Medicine and Health* 41, 151-156. doi: 10.2149/tmh.2013-06.

Ueno, H., Tomari, K., Kikuchi, K., Kobori, S., and Miyazaki, M. (2015). The first report of Vibrio parahaemolyticus O10:K60 in Japan, a new combination of O and K serotypes strain isolated from a gastroenteritis patient. *Jpn. J. Infect. Dis.*. doi: 10.7883/yoken.JJID.2014.538.

Velazquez-Roman, J., León-Sicairos, N., de Jesus Hernández-Díaz, L., and Canizalez-Roman, A. (2014). Pandemic Vibrio parahaemolyticus O3:K6 on the American continent. *Front. Cell. Infect. Mi.* 3, 110. doi: 10.3389/fcimb.2013.00110.

Velazquez-Roman, J., Leon-Sicairos, N., Flores-Villasenor, H., Villafana-Rauda, S., and Canizalez-Roman, A. (2012). Association of Pandemic Vibrio parahaemolyticus O3:K6 Present in the Coastal Environment of Northwest Mexico with Cases of Recurrent Diarrhea between 2004 and 2010. *Appl. Environ. Microb.* 78, 1794-1803. doi: 10.1128/AEM.06953-11.

Vongxay, K., Pan, Z., Zhang, X., Wang, S., Cheng, S., and Mei, L. (2008). Occurrence of pandemic clones of Vibrio parahaemolyticus isolates from seafood and clinical samples in a Chinese coastal province. *Foodborne Pathog. Dis.* 5, 127-34. doi: 10.1089/fpd.2007.0045.

Vuddhakul, V., Chowdhury, A., Laohaprertthisan, V., Pungrasamee, P., Patararungrong, N., and Thianmontri, P. (2000). Isolation of a pandemic O3:K6 clone of a Vibrio parahaemolyticus strain from environmental and clinical sources in Thailand. *Appl Environ Microbiol* 66, 2685-9.

Wootipoom, N., Bhoopong, P., Pomwised, R., Nishibuchi, M., Ishibashi, M., and Vuddhakul, V. (2007). A decrease in the proportion of infections by pandemic Vibrio parahaemolyticus in Hat Yai Hospital, southern Thailand. *J. Med. Microbiol.* 56, 1630-8. doi: 10.1099/jmm.0.47439-0.

Yan-yan, FAN., Min, ZHU., Xin-rong, SHANG., Mei, WANG., Yan-fei, HUANG and Hai-tong, G U., et al. (2013). Virulence characteristics and multilocus sequence type of Vibrio parahaemolyticus isolated from clinic. *Chin J Lab Med* 36, 548-52. (In Chinese). doi: 10.3760/cma.j.issn.1009-9158.2013.06.016.

Zavala-Norzagaray, A.A., Aguirre, A.A., Velazquez-Roman, J., Flores-Villasenor, H., Leon-Sicairos, N., and Ley-Quinonez, C.P. (2015). Isolation, characterization, and antibiotic resistance of Vibrio spp. in sea turtles from Northwestern Mexico. *Front. Microbiol.* 6, 635. doi: 10.3389/fmicb.2015.00635.
