# Supplementary material for: Sero-Prevalence and Genetic Diversity of Pandemic V. parahaemolyticus Strains Occurring at a Global Scale
Source: Front Microbiol. 2016 Apr 22;7:567. doi: 10.3389/fmicb.2016.00567 (PMC4840284; doi:10.3389/fmicb.2016.00567)
Supplement: Supplementary file 2 [file Table2.DOCX]

**Table S2. Epidemiological profile of** **185 isolates associated with STs of the pandemic clone and/or CC3**

| **Id** | **Isolate** | **Country** | **Year** | **Serotype** | **Source** | **Pandemic trait** | **STs** | **Reference** |
| --- | --- | --- | --- | --- | --- | --- | --- | --- |
| 1 | Unassigned | China | 2007-2012 | O3:K6 | clinical | yes | ST3 | (Li et al., 2014) |
| 2 | Unassigned | China | 2007-2012 | O1:KUT | clinical | yes | ST3 | (Li et al., 2014) |
| 3 | Unassigned | China | 2007-2012 | O1:K36 | clinical | yes | ST3 | (Li et al., 2014) |
| 4 | Unassigned | China | 2007-2012 | O4:K68 | clinical | yes | ST3 | (Li et al., 2014) |
| 5 | Unassigned | China | 2007-2012 | O5:K68 | clinical | yes | ST3 | (Li et al., 2014) |
| 6 | Unassigned | China | 2007-2012 | O1:K25 | clinical | yes | ST3 | (Li et al., 2014) |
| 7 | ATCC BAA-241 | India | 1998 | O4:K68 | clinical | yes | ST3 | (Ansaruzzaman et al., 2008) |
| 8 | AN-5034 | Bangladesh | 1998 | O4:K68 | clinical | yes | ST3 | (Gonzalez-Escalona et al., 2008) |
| 9 | VPHY145 | Thailand | 1999 | O4:K68 | clinical | yes | ST3 | (Gonzalez-Escalona et al., 2008) |
| 10 | Unassigned | Thailand | 1999 | O1:K25 | clinical | yes | ST3 | (Laohaprertthisan et al., 2003) |
| 11 | 056-01 | Peru | 2001 | O3:K6 | clinical | yes | ST3 | (Gavilan et al., 2013) |
| 12 | 004-02 | Peru | 2002 | O3:K6 | clinical | yes | ST3 | (Gavilan et al., 2013) |
| 13 | 038-03 | Peru | 2003 | O3:K6 | clinical | yes | ST3 | (Gavilan et al., 2013) |
| 14 | 205-05 | Peru | 2005 | O1:KUT | clinical | yes | ST3 | (Gavilan et al., 2013) |
| 15 | 275-99 | Peru | 1999 | O3:K58 | clinical | yes | ST3 | (Gavilan et al., 2013) |
| 16 | 276-99 | Peru | 1999 | O3:K6 | clinical | yes | ST3 | (Gavilan et al., 2013) |
| 17 | 304-07 | Peru | 2007 | O3:K30 | clinical | yes | ST3 | (Gavilan et al., 2013) |
| 18 | 330-00 | Peru | 2000 | O3:K6 | clinical | yes | ST3 | (Gavilan et al., 2013) |
| 19 | 784-98 | Peru | 1998 | O3:K6 | clinical | yes | ST3 | (Gavilan et al., 2013) |
| 20 | 369-07 | Peru | 2007 | O3:KUT | clinical | yes | ST3 | (Gavilan et al., 2013) |
| 21 | 301-07 | Peru | 2007 | O1:KUT | clinical | yes | ST3 | (Gavilan et al., 2013) |
| 22 | 906-97 | Peru | 1997 | O3:K6 | clinical | yes | ST3 | (Gavilan et al., 2013) |
| 23 | PMC50.7 | Chile | 2007 | O3:K6 | clinical | yes | ST3 | (Harth et al., 2009) |
| 24 | Unassigned | China | 2005-2008 | O1:KUT | environmental | yes | ST3 | (Chao et al., 2009) |
| 25 | V323 | China | 2006 | O1:KUT | clinical | yes | ST3 | (Chao et al., 2009) |
| 26 | V275 | China | 2006 | O1:K36 | clinical | yes | ST3 | (Chao et al., 2009) |
| 27 | Unassigned | China | 2005-2008 | O1:K25 | clinical | yes | ST3 | (Chao et al., 2009) |
| 28 | V377 | China | 2008 | O1:K56 | clinical | yes | ST3 | (Chao et al., 2009) |
| 29 | V504 | China | 2009 | O3:K6 | clinical | yes | ST3 | (Chao et al., 2009) |
| 30 | V283 | China | 2007 | O3:K25 | clinical | yes | ST3 | (Chao et al., 2009) |
| 31 | V258 | China | 2006 | O3:K68 | clinical | yes | ST3 | (Chao et al., 2009) |
| 32 | Unassigned | China | 2005-2008 | O4:K48 | clinical | yes | ST3 | (Chao et al., 2009) |
| 33 | Unassigned | China | 2005-2008 | O4:K68 | clinical | yes | ST3 | (Chao et al., 2009) |
| 34 | S061_1262 | Singapore | 1998 | O3:K6 | clinical | yes | ST3 | (Han et al., 2008) |
| 35 | S062_1263 | Singapore | 1998 | O6:K18 | clinical | yes | ST3 | (Han et al., 2008) |
| 36 | S063_1346 | China | 1998 | O1:K25 | clinical | yes | ST3 | (Han et al., 2008) |
| 37 | S064_1347 | China | 1998 | O3:K6 | clinical | yes | ST3 | (Han et al., 2008) |
| 38 | S065_1267 | China | 1998 | O1:K25 | clinical | yes | ST3 | (Han et al., 2008) |
| 39 | S066_1152 | China | 1997 | O3:K6 | clinical | yes | ST3 | (Han et al., 2008) |
| 40 | S067_1153 | China | 1997 | O3:K6 | clinical | yes | ST3 | (Han et al., 2008) |
| 41 | S068_1155 | China | 1997 | O3:K6 | clinical | yes | ST3 | (Han et al., 2008) |
| 42 | S071_1247 | Bangladesh | 1998 | O1:KUT | clinical | yes | ST3 | (Han et al., 2008) |
| 43 | S072_1248 | Bangladesh | 1998 | O3:K6 | clinical | yes | ST3 | (Han et al., 2008) |
| 44 | S074_1139 | China | 1997 | O3:K6 | clinical | yes | ST3 | (Han et al., 2008) |
| 45 | S075_1227 | China | 1999 | O3:K6 | clinical | yes | ST3 | (Han et al., 2008) |
| 46 | S079_1221 | Indonesia | 1997 | O3:K6 | clinical | yes | ST3 | (Han et al., 2008) |
| 47 | S077_1229 | China | 1997 | O3:K6 | clinical | yes | ST3 | (Han et al., 2008) |
| 48 | S081_1203 | Korea | 1997-1998 | O3:K6 | clinical | yes | ST3 | (Han et al., 2008) |
| 49 | S073_1023 | Thailand | 1997 | O3:K6 | clinical | yes | ST3 | (Han et al., 2008) |
| 50 | S083_1249 | Japan | 1998 | O3:K6 | clinical | yes | ST3 | (Han et al., 2008) |
| 51 | S084_1172 | Singapore | 1996 | O3:K6 | clinical | yes | ST3 | (Han et al., 2008) |
| 52 | S094_1456 | Thailand | 1996 | O3:K6 | clinical | yes | ST3 | (Han et al., 2008) |
| 53 | VPHY191 | Thailand | 1999 | O1:K25 | clinical | yes | ST3 | (Han et al., 2008) |
| 54 | S087_1264 | Singapore | 1998 | O4:K68 | clinical | yes | ST3 | (Han et al., 2008) |
| 55 | S090_1362 | China | 1999 | O4:K68 | clinical | yes | ST3 | (Han et al., 2008) |
| 56 | S091_1377 | India | 1999 | O4:K68 | clinical | yes | ST3 | (Han et al., 2008) |
| 57 | S095_1301 | China | 1996 | O3:K6 | clinical | yes | ST3 | (Han et al., 2008) |
| 58 | ICDC-VP77 | China | 2003 | O3:K6 | clinical | yes | ST3 | (Han et al., 2008) |
| 59 | ICDC-VP87 | China | 2004 | O3:K6 | clinical | yes | ST3 | (Han et al., 2008) |
| 60 | ICDC-VP88 | China | 2005 | O3:K6 | clinical | yes | ST3 | (Han et al., 2008) |
| 61 | ICDC-VP133 | China | 2007 | O3:K6 | clinical | yes | ST3 | (Han et al., 2008) |
| 62 | ICDC-VP53 | China | 2005 | O3:K6 | clinical | yes | ST3 | (Han et al., 2008) |
| 63 | ZJ3 | China | 2003 | O1:KUT | clinical | yes | ST3 | (Vongxay et al., 2008) |
| 64 | ZJ17 | China | 2003 | O3:K6 | clinical | yes | ST3 | (Vongxay et al., 2008) |
| 65 | IB3892 | Mozambique | 2004 | O3:K6 | clinical | yes | ST3 | (Ansaruzzaman et al., 2008) |
| 66 | IB3887 | Mozambique | 2004 | O4:K68 | clinical | yes | ST3 | (Ansaruzzaman et al., 2008) |
| 67 | 2888339 | Spain | 2004 | O3:K6 | clinical | yes | ST3 | (Martinez-Urtaza et al., 2005) |
| 68 | BA2 | China | 2008 | O3:K6 | clinical | yes | ST3 | (Ju et al., 2015) |
| 69 | GM6 | China | 2008 | O4:K68 | clinical | yes | ST3 | (Ju et al., 2015) |
| 70 | W1 | China | 2006 | O1:K25 | clinical | yes | ST3 | (Ju et al., 2015) |
| 71 | W50 | China | 2007 | O11:K36 | clinical | yes | ST3 | (Ju et al., 2015) |
| 72 | VP17MD | USA | 2012 | O3:K6 | clinical | yes | ST3 | pubMLST database |
| 73 | 222 | Ecuador | 1999 | O3:K6 | clinical | yes | ST3 | (Ellingsen et al., 2008) |
| 74 | 227 | Norway (probably) | 2002 | O5:K68 | clinical | yes | ST3 | (Ellingsen et al., 2008) |
| 75 | AO-24491 | Bangladesh | 1999 | O1:K25 | clinical | yes | ST3 | (Nair et al., 2007) |
| 76 | ATCC BAA-242 | India | 1998 | O1:KUT | clinical | yes | ST3 | (Nair et al., 2007) |
| 77 | VP81 | India | 1996 | O3:K6 | clinical | yes | ST3 | (Nair et al., 2007) |
| 78 | VPKX (RIMD 2210633) | Japan | 1996 | O3:K6 | clinical | yes | ST3 | (Gonzalez-Escalona et al., 2008) |
| 79 | PMA109.5 | Chile | 2005 | O3:K6 | Environment | yes | ST3 | (Gonzalez-Escalona et al., 2008) |
| 80 | KXV-641 | Japan | 1998 | O1:K25 | clinical | yes | ST3 | (Gonzalez-Escalona et al., 2008) |
| 81 | VP2 | Korea | 1998 | O3:K6 | clinical | yes | ST27 | (Gonzalez-Escalona et al., 2008) |
| 82 | BAC-98-3374 | USA | 1998 | O3:K6 | clinical | yes | ST42 | (Gonzalez-Escalona et al., 2008) |
| 83 | AP-11243 | Bangladesh | 2000 | O1:KUT | clinical | yes | ST51 | (Gonzalez-Escalona et al., 2008) |
| 84 | 220 | Ecuador | 1999 | O3:K6 | clinical | yes | ST71 | (Ellingsen et al., 2008) |
| 85 | 225 | Thailand | 2002 | O3:KUT | clinical | yes | ST72 | (Ellingsen et al., 2008) |
| 86 | V327 | China | 2007 | O1:K26 | clinical | yes | ST192 | (Chao et al., 2009) |
| 87 | V506 | China | 2009 | unknown | clinical | yes | ST227 | (Chao et al., 2009) |
| 88 | P202 | China | 2007 | O4: K8 | clinical | yes | ST283 | (Chen et al., 2012) |
| 89 | P15 | China | 2006 | O4:KUT | clinical | yes | ST301 | (Chen et al., 2012) |
| 90 | P43 | China | 2007 | O4:KUT | clinical | yes | ST302 | (Chen et al., 2012) |
| 91 | F6 | China | 2006 | O1:KUT | environmental | yes | ST305 | (Chen et al., 2012) |
| 92 | V-12 | China | 2008 | O1:K25 | clinical | yes | ST305 | (Chen et al., 2012) |
| 93 | 82 | China | 2010 | unknown | clinical | yes | ST431 | (Yan-yan et al., 2013) |
| 94 | SH11VP48 | China | 2011 | O3:K6 | clinical | yes | ST435 | pubMLST database |
| 95 | Hangzhou2010-599 | China | 2012 | O3:K6 | clinical | yes | ST672 | pubMLST database |
| 96 | V588 | China | 2009 | O1:K33 | environmental | no | ST266 | pubMLST database |
| 97 | Vp864 | China | 2007 | unknown | clinical | unknown | ST557 | pubMLST database |
| 98 | S092_1291 | China | 1996 | unknown | clinical | unknown | ST3 | pubMLST database |
| 99 | VP81 | India | 1996 | O3:K6 | clinical | unknown | ST3 | pubMLST database |
| 100 | VP86 | India | 1996 | O3:K6 | clinical | unknown | ST3 | pubMLST database |
| 101 | VP155 | India | 1996 | O3:K6 | clinical | unknown | ST3 | pubMLST database |
| 102 | VP96 | India | 1996 | O3:K6 | clinical | unknown | ST3 | pubMLST database |
| 103 | VP208 | India | 1997 | O3:K6 | clinical | unknown | ST3 | pubMLST database |
| 104 | AN-8373 | Bangladesh | 1998 | O3:K6 | clinical | unknown | ST3 | pubMLST database |
| 105 | JYKVP6 | Japan | 1998 | O3:K6 | clinical | unknown | ST3 | pubMLST database |
| 106 | TX2103 | USA | 1998 | O3:K6 | clinical | unknown | ST3 | pubMLST database |
| 107 | BAC-98-3372 | USA | 1998 | O3:K6 | clinical | unknown | ST3 | pubMLST database |
| 108 | BAC-98-4092 | USA | 1998 | O3:K6 | clinical | unknown | ST3 | pubMLST database |
| 109 | AN-16000 | Bangladesh | 1998 | O1:KUT | clinical | unknown | ST3 | pubMLST database |
| 110 | PMA 37.5 | Chile | 2005 | O3:K6 | environmental | unknown | ST3 | pubMLST database |
| 111 | PMA 109.5 | Chile | 2005 | O3:K6 | environmental | unknown | ST3 | pubMLST database |
| 112 | AN-2189 | Bangladesh | 1998 | O4:K68 | clinical | unknown | ST3 | pubMLST database |
| 113 | ATCC BAA-239 | India | 1996 | O3:K6 | clinical | unknown | ST3 | pubMLST database |
| 114 | ATCC BAA-238 | India | 1996 | O3:K6 | clinical | unknown | ST3 | pubMLST database |
| 115 | AN-7410 | Bangladesh | 1998 | O3:K6 | clinical | unknown | ST3 | pubMLST database |
| 116 | AN-11790 | Bangladesh | 1998 | O4:K68 | clinical | unknown | ST3 | pubMLST database |
| 117 | 226 | Thailand | 2002 | O3:KUT | clinical | No | ST3 | pubMLST database |
| 118 | FIHES98V1-32-4 | Japan | 1998 | O3:K6 | clinical | No | ST3 | pubMLST database |
| 119 | V197 | China | 2007 | O3:K6 | environmental | No | ST3 | pubMLST database |
| 120 | VP132 | Thailand | 2003 | O3:K46 | environmental | unknown | ST3 | pubMLST database |
| 121 | VP158 | Thailand | 2003 | O1:KUT | environmental | unknown | ST3 | pubMLST database |
| 122 | VP551 | USA | 2007 | unknown | environmental | unknown | ST3 | pubMLST database |
| 123 | 571 | USA | 2007 | unknown | environmental | unknown | ST3 | pubMLST database |
| 124 | 605 | USA | 2007 | unknown | environmental | unknown | ST3 | pubMLST database |
| 125 | 658 | USA | 2007 | unknown | environmental | No | ST3 | pubMLST database |
| 126 | 668 | USA | 2007 | unknown | environmental | unknown | ST3 | pubMLST database |
| 127 | 743 | USA | 2007 | unknown | environmental | No | ST3 | pubMLST database |
| 128 | VP747 | USA | 2007 | unknown | environmental | unknown | ST3 | pubMLST database |
| 129 | 752 | USA | 2007 | unknown | environmental | unknown | ST3 | pubMLST database |
| 130 | 782 | USA | 2007 | unknown | environmental | unknown | ST3 | pubMLST database |
| 131 | 783 | USA | 2007 | unknown | environmental | unknown | ST3 | pubMLST database |
| 132 | 861 | USA | 2007 | unknown | environmental | unknown | ST3 | pubMLST database |
| 133 | 863 | USA | 2007 | unknown | environmental | No | ST3 | pubMLST database |
| 134 | 949 | USA | 2007 | unknown | environmental | unknown | ST3 | pubMLST database |
| 135 | BE98-2029 | USA | 1998 | unknown | clinical | No | ST3 | pubMLST database |
| 136 | WX1465 | China | 2014 | O3:K6 | clinical | unknown | ST3 | pubMLST database |
| 137 | VP16MD | USA | 2012 | unknown | clinical | unknown | ST3 | pubMLST database |
| 138 | VP17MD | USA | 2012 | unknown | clinical | unknown | ST3 | pubMLST database |
| 139 | VP18MD | USA | 2012 | unknown | clinical | unknown | ST3 | pubMLST database |
| 140 | Peru-466 | Peru | 1996 | unknown | clinical | unknown | ST3 | pubMLST database |
| 141 | HY20 | China | 2007 | O11:K36 | clinical | unknown | ST3 | pubMLST database |
| 142 | HY19 | China | 2007 | O11:K36 | clinical | No | ST3 | pubMLST database |
| 143 | GM8 | China | 2008 | O4:K68 | clinical | No | ST3 | pubMLST database |
| 144 | V-213 | China | 2012 | O4:K68 | clinical | No | ST3 | pubMLST database |
| 145 | Peru-288 | Peru | 2001 | unknown | clinical | unknown | ST3 | pubMLST database |
| 146 | VIP4 | China | 2007 | unknown | clinical | unknown | ST3 | pubMLST database |
| 147 | VIP-0439 | China | 2008 | unknown | clinical | unknown | ST3 | pubMLST database |
| 148 | B-265 | Mozambique | 2004 | unknown | clinical | unknown | ST3 | pubMLST database |
| 149 | EKP-028 | Bangladesh | 2008 | unknown | environmental | unknown | ST3 | pubMLST database |
| 150 | WX1472 | China | 2014 | O3:K6 | clinical | unknown | ST3 | pubMLST database |
| 151 | WX1478 | China | 2014 | O3:K6 | clinical | unknown | ST3 | pubMLST database |
| 152 | WX14116 | China | 2014 | O3:K6 | clinical | unknown | ST3 | pubMLST database |
| 153 | CDC_K5010_1 | USA | 2006 | O1:KUT | clinical | unknown | ST3 | pubMLST database |
| 154 | CDC_K5058 | USA | 2007 | O3:K6 | clinical | unknown | ST3 | pubMLST database |
| 155 | CDC_K5528 | USA | 2007 | O4:K68 | clinical | unknown | ST3 | pubMLST database |
| 156 | CAIM 729 | USA | 1998 | O3:K6 | clinical | unknown | ST3 | pubMLST database |
| 157 | CAIM 1400 | Mexico | 2004 | O3:K6 | clinical | unknown | ST3 | pubMLST database |
| 158 | CAIM 1474 | Mexico | 2004 | O3:K6 | clinical | unknown | ST3 | pubMLST database |
| 159 | CAIM 1477 | Mexico | 2004 | O3:K6 | clinical | unknown | ST3 | pubMLST database |
| 160 | CAIM 1490 | Mexico | 2004 | O3:K6 | clinical | unknown | ST3 | pubMLST database |
| 161 | CAIM 1693 | Mexico | 2004 | O3:K6 | clinical | unknown | ST3 | pubMLST database |
| 162 | CICESE-170 | Mexico | 1998 | O3:K6 | clinical | unknown | ST3 | pubMLST database |
| 163 | CICESE-171 | Mexico | 1998 | O3:K6 | clinical | unknown | ST3 | pubMLST database |
| 164 | CICESE -173 | Mexico | 1998 | O3:K6 | clinical | unknown | ST3 | pubMLST database |
| 165 | CICESE -174 | Mexico | 1998 | O3:K6 | clinical | unknown | ST3 | pubMLST database |
| 166 | CICESE -175 | Mexico | 1998 | O3:K6 | clinical | unknown | ST3 | pubMLST database |
| 167 | CICESE -176 | Mexico | 1998 | O3:K6 | clinical | unknown | ST3 | pubMLST database |
| 168 | CICESE -177 | Mexico | 1998 | O3:K6 | clinical | unknown | ST3 | pubMLST database |
| 169 | CICESE -178 | Mexico | 1998 | O3:K6 | clinical | unknown | ST3 | pubMLST database |
| 170 | CICESE -179 | Mexico | 1998 | O3:K6 | clinical | unknown | ST3 | pubMLST database |
| 171 | CICESE -180 | Mexico | 1998 | O3:K6 | clinical | unknown | ST3 | pubMLST database |
| 172 | CICESE -181 | Mexico | 1998 | O3:K6 | clinical | unknown | ST3 | pubMLST database |
| 173 | CICESE -182 | Mexico | 1998 | O3:K6 | clinical | unknown | ST3 | pubMLST database |
| 174 | CICESE -183 | Mexico | 1998 | O3:K6 | clinical | unknown | ST3 | pubMLST database |
| 175 | CICESE -184 | Mexico | 1998 | O3:K6 | clinical | unknown | ST3 | pubMLST database |
| 176 | CICESE -186 | Mexico | 1999 | O3:K6 | clinical | unknown | ST3 | pubMLST database |
| 177 | CICESE -187 | Mexico | 2000 | O3:K6 | clinical | unknown | ST3 | pubMLST database |
| 178 | CICESE -188 | Mexico | 2009 | O3:K6 | clinical | unknown | ST3 | pubMLST database |
| 179 | CICESE -273 | Mexico | 2012 | O3:K6 | environmental | unknown | ST3 | pubMLST database |
| 180 | V94 | China | 2007 | O3:K6 | environmental | no | ST2 | pubMLST database |
| 181 | V548 | China | 2009 | O3:K6 | environmental | no | ST220 | pubMLST database |
| 182 | F10-105 | China | 2010 | O4:K68 | clinical | unknown | ST787 | pubMLST database |
| 183 | VP25 | China | 2012 | unknown | clinical | unknown | ST886 | pubMLST database |
| 184 | CICESE -185 | Mexico | 1998 | O3:K6 | clinical | unknown | ST1139 | pubMLST database |
| 185 | V-606-07 | Chile | 2007 | unknown | clinical | unknown | ST1172 | pubMLST database |

**REFERENCES:**

Ansaruzzaman, M., Chowdhury, A., Bhuiyan, N.A., Sultana, M., Safa, A., and Lucas, M. (2008). Characteristics of a pandemic clone of O3 : K6 and O4 : K68 Vibrio parahaemolyticus isolated in Beira, Mozambique. *J. Med. Microbiol.* 57, 1502-7. doi: 10.1099/jmm.0.2008/004275-0.

Chao, G., Jiao, X., Zhou, X., Yang, Z., Huang, J., and Pan, Z. (2009). Serodiversity, pandemic O3:K6 clone, molecular typing, and antibiotic susceptibility of foodborne and clinical Vibrio parahaemolyticus isolates in Jiangsu, China. *Foodborne Pathog. Dis.* 6, 1021-8. doi: 10.1089/fpd.2009.0295.

Chen, W., Xie, Y., Xu, J., Wang, Q., Gu, M., and Yang, J. (2012). Molecular typing of Vibrio parahaemolyticus isolates from the middle-east coastline of China. *Int. J. Food Microbiol.* 153, 402-12. doi: 10.1016/j.ijfoodmicro.2011.12.001.

Ellingsen, A.B., Jorgensen, H., Wagley, S., Monshaugen, M., and Rorvik, L.M. (2008). Genetic diversity among Norwegian Vibrio parahaemolyticus. *J. Appl. Microbiol.* 105, 2195-202. doi: 10.1111/j.1365-2672.2008.03964.x.

Gavilan, R.G., Zamudio, M.L., and Martinez-Urtaza, J. (2013). Molecular epidemiology and genetic variation of pathogenic Vibrio parahaemolyticus in Peru. *PLoS Negl Trop Dis* 7, e2210. doi: 10.1371/journal.pntd.0002210.

Gonzalez-Escalona, N., Martinez-Urtaza, J., Romero, J., Espejo, R.T., Jaykus, L.A., and DePaola, A. (2008). Determination of molecular phylogenetics of Vibrio parahaemolyticus strains by multilocus sequence typing. *J. Bacteriol.* 190, 2831-40. doi: 10.1128/JB.01808-07.

Han, H., Wong, H.C., Kan, B., Guo, Z., Zeng, X., and Yin, S. (2008). Genome plasticity of Vibrio parahaemolyticus: microevolution of the 'pandemic group'. *BMC Genomics* 9, 570. doi: 10.1186/1471-2164-9-570.

Harth, E., Matsuda, L., Hernandez, C., Rioseco, M.L., Romero, J., and Gonzalez-Escalona, N. (2009). Epidemiology of Vibrio parahaemolyticus outbreaks, southern Chile. *Emerg. Infect. Dis.* 15, 163-8.

Ju, C., Yu, M., Huang, R., Luo, J., and Duan, Y. (2015). Genetic characterization of Vibrio parahaemolyticus O3: K6 serovariant isolated in Shenzhen. *Zhonghua Yu Fang Yi Xue Za Zhi* 49, 21-5.

Laohaprertthisan, V., Chowdhury, A., Kongmuang, U., Kalnauwakul, S., Ishibashi, M., and Matsumoto, C. (2003). Prevalence and serodiversity of the pandemic clone among the clinical strains of Vibrio parahaemolyticus isolated in southern Thailand. *Epidemiol. Infect.* 130, 395-406.

Li, Y., Xie, X., Shi, X., Lin, Y., Qiu, Y., and Mou, J. (2014). Vibrio parahaemolyticus, Southern Coastal Region of China, 2007-2012. *Emerg. Infect. Dis.* 20, 685-8. doi: 10.3201/eid2004.130744.

Martinez-Urtaza, J., Simental, L., Velasco, D., DePaola, A., Ishibashi, M., and Nakaguchi, Y. (2005). Pandemic Vibrio parahaemolyticus O3:K6, Europe. *Emerg. Infect. Dis.* 11, 1319-20. doi: 10.3201/eid1108.050322.

Nair, G.B., Ramamurthy, T., Bhattacharya, S.K., Dutta, B., Takeda, Y., and Sack, D.A. (2007). Global dissemination of Vibrio parahaemolyticus serotype O3:K6 and its serovariants. *Clin. Microbiol. Rev.* 20, 39-48. doi: 10.1128/CMR.00025-06.

Vongxay, K., Pan, Z., Zhang, X., Wang, S., Cheng, S., and Mei, L. (2008). Occurrence of pandemic clones of Vibrio parahaemolyticus isolates from seafood and clinical samples in a Chinese coastal province. *Foodborne Pathog. Dis.* 5, 127-34. doi: 10.1089/fpd.2007.0045.

Yan-yan, FAN., Min, ZHU., Xin-rong, SHANG., Mei, WANG., Yan-fei, HUANG and Hai-tong, G U., et al (2013). Virulence characteristics and multilocus sequence type of Vibrio parahaemolyticus isolated from clinic. *Chin J Lab Med* 36, 548-52. (In Chinese). doi: 10.3760/cma.j.issn.1009-9158.2013.06.016.
